# Clinical features of Infantile Epileptic Spasms Syndrome: a systematic review

**DOI:** 10.1186/s13023-026-04229-1

**Published:** 2026-02-02

**Authors:** Xiao Meng, Danielle S. Takacs, Yashaswini Kelagere, Zihan Zhang, Sanjanaa Seshadri, Jiyeon Cha, Ming Li, Lei-Shih Chen

**Affiliations:** 1 Department of Health Behavior, School of Public Health, Texas A&M University, College Station, USA; 2 Division of Neurology and Developmental Neuroscience, Department of Pediatrics, Texas Children’s Hospital, Baylor College of Medicine, Houston, USA; 3 Department of Health Sciences, Towson University, Maryland, USA

**Keywords:** Infantile Epileptic Spasms Syndrome, Clinical features, Pediatricians, Systematic review

## Abstract

**Introduction:**

Infantile Epileptic Spasms Syndrome (IESS) is a severe epilepsy in children between one month and two years of age. Urgent referral of possible IESS cases from pediatricians and family physicians who care for children to neurologists for diagnosis and treatment has been shown to significantly improve patient health outcomes. Yet, a comprehensive list of IESS clinical features is lacking. We conducted a systematic review of studies to report IESS clinical features.

**Methods:**

Articles from the MEDLINE Complete and Embase databases were searched from the earliest years of databases availability (1979) to July 31, 2024. We followed the Preferred Reporting for Systematic Reviews and Meta-Analysis guidelines to conduct this systematic review with 140 articles, all of which were descriptive studies.

**Results:**

We identified 25 clinical features among the 3,786 children with IESS who were included in 140 studies. No single clinical descriptor was reported in over half of the children. The six most commonly reported clinical features included flexor spasms (48.28%), mixed spasms (30.19%), extensor spasms (10.49%), symmetric spasms (8.49%), asymmetric/focal spasms (6.50%), and head nodding (2.48%).

**Conclusions:**

There were a wide variety of IESS presentation descriptions. This review will help future researchers to develop an IESS screening tool that can assist pediatricians and family physicians who care for children in making urgent referrals.

**Supplementary Information:**

The online version contains supplementary material available at 10.1186/s13023-026-04229-1.

## Introduction

Infantile Epileptic Spasms Syndrome (IESS), historically referred to as infantile or epileptic spasms, is a term used to describe a severe form of epilepsy presenting in children that typically occurs between one month and two years of age [1]. IESS includes West syndrome, which is defined as a triad of epileptic spasms, hypsarrhythmia on an electroencephalogram (EEG), and developmental delay or regression [2]. In the United States (U.S.), approximately 2000 to 2500 new IESS cases are reported annually [3]. Children with IESS can experience up to hundreds of seizures daily that can lead to severe cognitive and developmental disabilities [4, 5]. Nevertheless, early referral, diagnosis, and treatment of IESS—ideally within 7 days of symptom onset [6]—have been shown to significantly improve health outcomes. These outcomes include a reduction in developmental delays, a decrease in intensive healthcare requirements, and an increase in the possibility of becoming seizure-free at the 2-weeks and 3 months post-treatment mark, with a potential minimization of a long-term seizure burden of children with IESS [7–10]. 

Clinical features are critical for medical decision-making, particularly for IESS, a complex condition often initially identified through the observation of subtle spasms [7]. Currently, the American Academy of Pediatrics (AAP) provides a list of infantile spasms symptoms [11]. Yet, the list only includes 5 symptoms (i.e., body stiffening, back arching, jerking at the tummy, arm raising, and wide-eyed blinks or eye rolling) [11], which makes it challenging for pediatricians and family physicians who care for children to promptly identify IESS signs from limited observations and parental reports. This challenge often causes significant delays in the referral, diagnosis, and treatment of children with IESS. Research has found that the average delay in diagnosis and treatment was 1 month and might exceed 3 months [6, 12–14]. 

The goal of this systematic review literature is to provide a comprehensive listing of the IESS symptoms reported in the literature and an assessment of the methodological quality of the reviewed studies. The findings of our study will (1) assist pediatricians and family physicians who often have limited training in neurology and epilepsy, in recognizing IESS-related clinical features at early stages in high-risk patients to facilitate timely referrals to pediatric neurologists for diagnosis and treatment [15–17] and (2) help parents and caregivers who observe their children with abnormal movements to acknowledge those signs and report them to pediatricians and family physicians who care for children.

## Methods

### Database search strategy

We followed the Preferred Reporting for Systematic Reviews and Meta-Analysis (PRISMA) guidelines to conduct this systematic review [18]. Specifically, we systematically searched MEDLINE Complete (EBSCO) and Embase to identify articles published from the earliest years of the databases (January 1, 1978) to July 31, 2024. We used the following medical terms as keywords in both headings and text words, and combined the keywords in all possible combinations: (1) keywords relating to IESS: “infantile spasms,” “West syndrome,” and “epileptic spasms,” and (2) keywords relating to clinical features: “symptom,” “characteristic,” “abnormal behavior,” “sign,” “feature,” and “presentation.” The systematic review team and a medical librarian contribute to the development of the search strategy and choosing which search terms were used to optimize for exhaustivity.

### Selection of studies

The inclusion criteria for the articles included in this systematic review were: (1) original studies published in English-language peer-reviewed journals, (2) children with IESS presenting between 1 month to 2 years of age, (3) studies reporting and describing the clinical features of IESS. Studies were excluded based on the following criteria: (1) reviews and conference abstracts, (2) non-peer-reviewed publications, (3) articles not published in English, and (4) articles including patients with IESS that did not describe clinical symptoms.

Two authors (Y.K. and S.S.) independently reviewed the titles and abstracts of all studies in the initial search and determined the need for a full-text review based on the study eligibility criteria. Rayyan QCRI was used for screening and duplicate entry elimination [19]. Full-text screening was independently completed by the same 2 authors using the study inclusion and exclusion criteria. Any disagreements were discussed, if necessary, with the involvement of the entire research team until a consensus was reached.

### Data extraction

Data from the studies included in this systematic review were independently extracted and organized into a matrix table (Supplemental Table 1) by 2 authors (X.M. and Y.K.). Information collected from each study included the authors’ names, publication year and country, sample size, patient sex and ethnicity, study design, diagnostic tools, patient age at symptom onset, patient age at diagnosis, and clinical features. Using Gwet’s AC1 [20], the inter-rater reliability was determined to be 0.90, which indicated a high level of consistency between the 2 authors. Disagreements and inconsistencies were discussed until agreement was reached by the entire research team.

### Study methodological quality assessment

To evaluate the methodological quality of studies included in this systemic review, a methodological quality score (MQS) was used that consisted of 5 assessment criteria with a possible score range of 2 to 10. The MQS assessment items included the evaluation of the sample size of the patients with IESS, patient sex, patient age at symptom onset and diagnosis, and study design (Table 1) and were developed by adapting the evaluation criteria from previous systematic reviews and literature [21–25]. For example, study designs were categorized into 3 types: descriptive study, analytical study, and controlled studies [25]. Table 1 presents the MQS criteria and summarizes the findings derived from the included studies. Studies with higher MQS scores denote higher level of methodological quality.

**Table 1:** Frequency distribution of methodological quality among reviewed articles (*n* = 140)

Methodological criterion	Description	Points	Frequency distribution	
			*n*	%
Sample size of patients with IESS	< 50	1	120	85.71
	≥ 50 and < 100	2	10	7.14
	≥ 100 and < 300	3	6	4.29
	≥ 300	4	4	2.86
Gender	Not reported	0	11	7.86
	Reported	1	129	92.14
Age at IESS symptom onset	Not reported	0	20	14.29
	Reported	1	120	85.71
Age at IESS diagnosis	Not reported	0	116	82.86
	Reported	1	24	17.14
Study design	Descriptive study	1	140	100%
	Analytical study	2	0	0
	Controlled study	3	0	0

Abbreviations: IESS = infantile epileptic spasms syndrome

## Results

### Search results

**Fig. 1:**
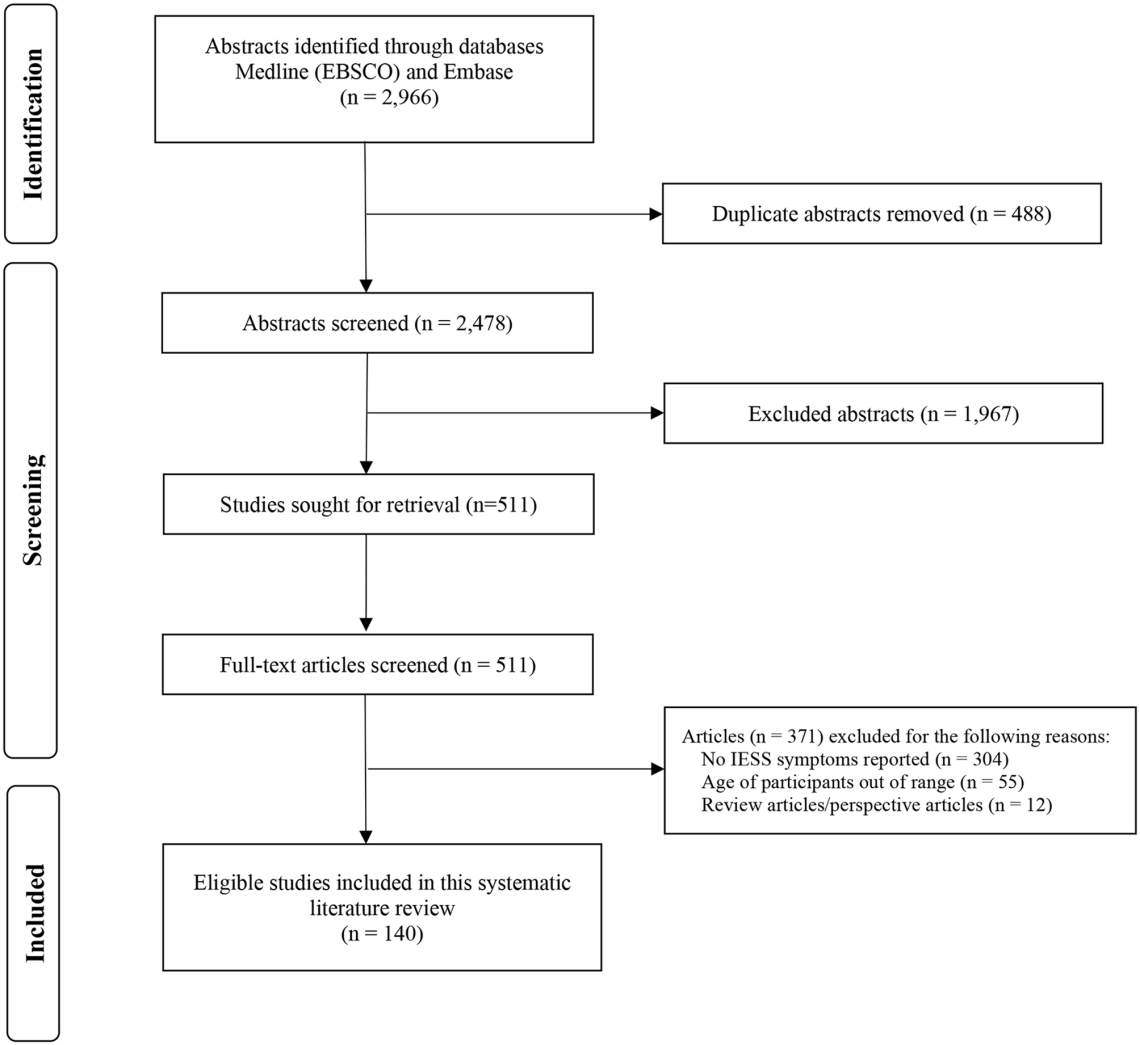
A PRISMA flow diagram illustrating the process of identifying, screening, and selecting articles included in this systematic review. Abbreviations: IESS = Infantile Epileptic Spasms Syndrome; PRISMA=Preferred Reporting Items for Systematic Reviews and Meta-Analyses

Figure 1 illustrates the article selection process in this systematic review. We first identified a total of 2966 articles through MEDLINE Complete (EBSCO) and Embase databases and then removed 488 duplicate articles. After screening the titles and abstracts of 2478 articles, a further 1967 articles were then excluded. Next, full-text reviews of 511 potentially eligible articles were carefully reviewed, and an additional 371 articles were excluded. Finally, a total of 140 publications were found to have met the eligibility criteria to be included in our analysis.

### Description of the studies (Supplemental table 1)

#### Study year, location, and design

The publication years of the 140 articles included in this systematic review ranged from 1979 to 2024 and varied by decade: 1979 (*n* = 2; 1.43%), 1980 to 1989 (*n* = 7; 5.77%), 1990 to 1999 (*n* = 22; 15.71%), 2000 to 2009 (*n* = 33; 23.57%), 2010 to 2019 (*n* = 52; 37.14%), and 2020 to 2024 (*n* = 24; 17.14%). While the majority of the included studies (96.43%) were conducted within a single country, 5 studies (3.57%) were conducted across multiple countries. Studies included data collected from 39 regions that included from the highest frequency to lowest frequency: Japan (*n* = 22; 15.71%), U.S. (*n* = 21; 15.00%), Italy (*n* = 15; 10.71%), China (*n* = 10; 7.14%), India (*n* = 9; 6.43%), Turkey (*n* = 9; 6.43%), France (*n* = 8; 5.71%), Canada (*n* = 7; 5.00%), South Korea (*n* = 5; 3.57%), Argentina (*n* = 5; 3.57%), Pakistan (*n* = 3; 2.14%), the United Kingdom (*n* = 3; 2.14%), Croatia (*n* = 2; 1.43%), Spain (*n* = 2; 1.43%), Germany (*n* = 2; 1.43%), and other countries (*n* = 24, 17.14%). All the articles we included in this review were descriptive studies focusing on the clinical features of IESS.

#### Patient number and characteristics

The total number of patients diagnosed with IESS in the articles included in this systematic review was 3786, but the number of patients in each article varied, ranging from 1 to 358. The majority of the reviewed studies (*n* = 130; 92.86%) reported fewer than 100 patients with IESS, and about half of the included studies reported a single case (*n* = 64; 45.71%). About half (*n* = 1,822; 48.12%) of the patients in the included studies were male, 1179 (31.14%) were female, and there were 785 (20.73%) patients for whom patient sex was not reported. Most of the included studies (*n* = 124; 88.57%) did not report patient ethnicity.

### Clinical features of IESS

All reviewed articles stated that the symptoms observed in patients were confirmed by clinicians. Twenty-five types of symptoms were reported in the 3786 patients included in the studies we reviewed, though most patients (*n* = 3257; 86.03%) had only one symptom reported. As shown in Table 2, the IESS symptom descriptions, from the highest frequency to the lowest frequency, included: flexor spasms (*n* = 1828; 48.28%), mixed (flexor/extensor) spasms (*n* = 1143; 30.19%), extensor spasms (*n* = 397; 10.49%), symmetric spasms (*n* = 321; 8.49%), asymmetric/focal spasms (*n* = 246; 6.50%), head nodding (*n* = 94; 2.48%), abnormal eye movement (*n* = 75; 1.98%), myoclonic jerks (*n* = 63; 1.66%), tonic spasms (*n* = 49; 1.29%), subtle spasms (*n* = 26; 0.69%), atypical spasms (*n* = 21; 0.55%), head deviation (*n* = 10; 0.26%), akinetic spasms (*n* = 8; 0.21%), associated crying (*n* = 6; 0.16%), behavior arrest (*n* = 5; 0.13%), facial movements/grimace (*n* = 5; 0.13%), unclear spasms (*n* = 5; 0.13%), acoustic hypersensitivity/startle (*n* = 3; 0.08%), frightened expression (*n* = 3; 0.08%), spasms of the corner of the mouth (*n* = 3; 0.08%), postural spasms (*n* = 2; 0.05%), vocalization (*n* = 2; 0.05%), opisthotonos (*n* = 1; 0.03%), hemi-body convulsions (*n* = 1; 0.03%), and swallowing and vomiting (*n* = 1; 0.03%).

**Table 2:** Number and percentages of clinical features of IESS among 3,786 patients from reviewed studies

Clinical features	Numbers of patients	Percentage (%)
Flexor spasms	1,828	48.28
Mixed (flexor/extensor) spasms	1,143	30.19
Extensor spasms	397	10.49
Symmetric spasms	321	8.49
Asymmetric/focal spasms	246	6.50
Head nodding	94	2.48
Abnormal eye movement (e.g., upward eye movement, eye deviation, nystagmoid movements, eyelid myoclonia, and eye fluttering/blinking)	75	1.98
Myoclonic jerks	63	1.66
Tonic spasms	49	1.29
Subtle spasms	26	0.69
Atypical spasms	21	0.55
Head deviation	10	0.26
Akinetic spasms	8	0.21
Associated crying	6	0.16
Behavioral arrest	5	0.13
Facial movements/grimace	5	0.13
Unclear spasms	5	0.13
Acoustic hypersensitivity/startle	3	0.08
Frightened expression	3	0.08
Spasms of the corner of the mouth	3	0.08
Postural spasms	2	0.05
Vocalization	2	0.05
Opisthotonos	1	0.03
Hemi-body convulsions	1	0.03
Swallowing and vomiting	1	0.03

Notes: Some patients may have more than one clinical feature

### Diagnostic tool

All studies (*n* = 140; 100%) reported using an EEG as an IESS diagnostic tool. Yet, most of the articles we reviewed did not specify the duration of the EEG (*n* = 130; 92.86%) or whether sleep data were recorded during EEG (*n* = 130; 92.86%). Only 10 articles (7.14%) that included 252 patients (6.66%) reported EEG duration, which ranged from 50 minutes to over 24 hours. Only 10 articles (7.14%) that included 395 patients (10.43%), stated that EEG recordings were obtained during sleep.

### IESS onset age and diagnosis age

Regarding the age of onset of IESS, 120 articles (85.71%) specifically reported the age of patients at the onset of IESS symptoms (*n* = 2972; 78.50%). The average reported onset age of symptoms was 6.02 months, with a range of 1 to 84 months. Regarding diagnosis age (confirmed with an EEG), only 24 articles (17.14%) with a total of 638 patients reported the age of the patient at IESS diagnosis. The average reported age at diagnosis confirmation was 10.13 months, with a range of 1 to 15 months.

### Methodological quality of the studies

The studies we reviewed were found to have a mean MQS score of 4.19, with scores ranging from 2 to 7 (Table 1) within the theoretical range of 2 to 10, with a higher MQS scores indicating a higher level of methodological quality. One of the studies with the highest MQS score was a study conducted by Gulati and colleagues [26] in India involving 310 cases of IESS (234 males, 76 females). The authors reported that all patients underwent interictal sleep EEG recordings and the median age of symptom onset was 5 months with a range of 2.5 to 7 months. The reported types of spasms were predominantly flexor spasms (78.39%), followed by mixed flexor/extensor spasms (11.29%) and extensor spasms (10.32%) [26].

Most studies reported patient sex (*n* = 128; 91.43%) and age at symptom onset (*n* = 120; 85.71%). Nevertheless, the majority of the studies that we reviewed reported fewer than 50 participants (*n* = 120; 85.71%) and did not report age at diagnosis (*n* = 116; 82.86%). For example, Fukuoka and colleagues [27] published a case report of a patient with IESS and severe developmental delay. The patient was male and 7 months of age at IESS symptom onset, but the age at diagnosis was not reported [27].

## Discussion

This systematic literature review reports all IESS clinical features documented in the literature. Based on reports of 3786 IESS patients described in 140 articles, we found a total of 25 clinical features. The 6 most common clinical features reported included flexor spasms (48.28%), mixed spasms (30.19%), extensor spasms (10.49%), symmetric spasms (8.49%), asymmetric/focal spasms (6.50%), and head nodding (2.48%). We also found 16 identified clinical features that occurred in less than 1% of reported IESS patients. Such diverse but low frequency clinical features present a significant challenge for pediatricians and family physicians who care for children in recognizing IESS symptoms and making referrals to pediatric neurologists for care. Additionally, it is important to highlight that no single clinical descriptor was reported in over half of the IESS patients, suggesting variability in how the clinical features of IESS and spasms movements were perceived and documented.

It is critical that pediatricians and family physicians who care for pediatric patients be able to recognize IESS clinical features and facilitate urgent referral for suspected IESS cases [6]. Delays in IESS referral and diagnosis increase the anxiety of parents of children with IESS, and studies have reported that some parents of children with IESS had to see multiple physicians before finding one willing to make the needed referral to a neurologists for care [6, 28]. To reduce parental distress and promote better patient care, timely IESS referrals and diagnoses should be prioritized. Studies have suggested that pediatricians and family physicians should receive additional training in epilepsy to recognize IESS faster [6, 13]. The 25 IESS clinical features identified in this systemic review can inform the development of continued education, such as presenting videos of various IESS spasms presentations, to help pediatricians and family physicians accurately recognize IESS symptoms and make timely referrals. Because clinical features may not be observed during a short medical appointment, obtaining videos of the abnormal movements of the child from their parents and caregivers may be needed to help pediatricians and family physicians accurately recognize symptoms [29]. 

Notably, most studies included in this systematic review did not provide information on EEG duration and whether sleep was recorded during EEG - details that carry significant implications for research and the clinical diagnosis of IESS. Obtaining sleep data during EEG and EEG duration are both critical factors in accurately diagnosing IESS, as they directly influence the ability of EEG to detect epileptiform abnormalities and capture clinical events [30, 31]. Failure to obtain sleep data from EEG and short duration EEG, such as most routine outpatient EEGs that are typically are less than 30 minutes in length, may miss epileptiform abnormalities, and be inadequate to capture the epileptic spasms of patients with IESS [30–33]. As such, it is imperative that future research reports both EEG duration and whether sleep was recorded during EEG to ensure diagnostic accuracy and scientific rigor.

The mean MQS scores of articles we reviewed suggests that the overall methodological quality level of this body of literature was low. Specifically, most studies were limited by a small sample size, with many reporting only a single case or a few cases. Because IESS is a rare disease, it is understandable that a small sample size may be reported in research. Yet, such small sample sizes may undermine the validity of the studies and pose challenges to the generalization of their findings. Furthermore, although most of the articles we included in this review report patient age at symptom onset, most did not specify patient age at diagnosis. This gap creates challenges in understanding the full timeline of the IESS diagnostic journey. In addition, all articles we reviewed employ descriptive studies. Compared to analytical and controlled studies, descriptive studies lack a comparison of critical factors such as the clinical features that differentiate IESS and non-IESS medical conditions. Accordingly, future research may need to employ more rigorous methodological approaches in this body of the literature.

The findings of this systematic literature are subject to several inherent limitations. Although IESS typically occurs between 1 month to 2 years of age, a few studies only reported an age range for all participants, without specifying the individual ages of each patient. Thus, our review might have included a small number of participants younger than 1 month or older than 2 years of age. Additionally, while it would be informative to report standard deviation and quartiles of patients with IESS in our review, we were unable to do so as the majority of the original studies we reviewed did not provide this information. Moreover, as there was no pre-existing gold standard for assessing the quality of the studies we reviewed, we developed the MQS by drawing on previous literature. Additionally, our search was limited to English-language literature only, which potentially excluded relevant articles published in other languages. Furthermore, though we used various strategies to search articles, a few studies might have been missed in this systematic literature review study. Lastly, it is possible that the same IESS symptoms were described with different descriptive terms in the articles we reviewed. Yet, we retained the original terms that were commonly used by pediatric providers to enhance the applicability of our findings in real-world clinical practice. Using pediatric provider-friendly language terms to describe IESS signs and symptoms can improve recognition and communication of IESS symptoms to practicing pediatricians and family physicians who care for children.

Despite these limitations, this systematic literature review has significant strengths and clinical implications. First, to the best of our knowledge, this is the first systematic literature review that has comprehensively reported IESS clinical features across a large number of studies. We identified all IESS clinical features observed in 3786 patients in 140 research studies across a broad timeline. Second, our review provides up-to-date, evidence-based information that expands the limited AAP’s IESS symptoms list and provides critically needed information that will help future researchers develop an IESS screening tool that will assist pediatricians and family physicians who care for children in recognizing IESS symptoms earlier. This tool can incorporate the identified clinical features and supplementary videos to support general practitioners and family physicians in accurately and promptly recognizing IESS. Third, our systematic review highlighted delays in IESS diagnoses. While numerous factors likely underlie delays in diagnosis, including limited parental awareness of IESS [34], a lack of access to pediatric neurologists [35], a paucity of pediatric neurologists in the U.S [36, 37], a long travel distance to pediatric neurology care [38], and health insurance restriction [39], this issue could also be addressed by increasing the IESS awareness of pediatricians and family physicians who care for children. Continuing education for pediatricians and family physicians, advocacy from professional associations such as the AAP, and recognition of available resources, such as those from the Infantile Spasms Action Network [40] may increase the IESS knowledge and awareness of pediatricians and family physicians who care for children and empower them to make timely referrals. Finally, there is a need for the application of more rigorous research methodologies in this body of the literature.

## Conclusion

This systematic literature review offers a comprehensive overview of the clinical features of IESS among 3786 patients across 140 research studies, highlighting considerable variability in how IESS symptoms present or are described in the literature. Our review also revealed a notable delay in IESS diagnosis. As pediatricians serve as the primary gatekeepers of young children’s health, a deeper understanding of IESS symptoms can enable them to make timely referrals to neurologists for specialized care. Furthermore, the insights from this review could guide future researchers in developing an IESS screening tool to assist pediatricians in facilitating urgent referrals. Lastly, given the generally low methodological quality of the studies we reviewed, we recommend that future research adopt more rigorous approaches to enhance the quality of this body of literature.

## Supplementary Information

Below is the link to the electronic supplementary material.

Supplementary Material 1

## Data Availability

Data supporting the findings of this study are included in the figure, tables, and supplementary materials.
