# Supplementary material for: Clinical features of Infantile Epileptic Spasms Syndrome: a systematic review
Source: Orphanet J Rare Dis. 2026 Feb 2;21:81. doi: 10.1186/s13023-026-04229-1 (PMC12955028; doi:10.1186/s13023-026-04229-1)
Supplement: Supplementary file 1 — Supplementary Material 1 [file 13023_2026_4229_MOESM1_ESM.docx]

**Clinical Features of Infantile Epileptic Spasms Syndrome: A Systematic Review**

| **SUPPLEMENTAL TABLE 1** Study Characteristics (n=140) | | | | | | | | | | |
| --- | --- | --- | --- | --- | --- | --- | --- | --- | --- | --- |
| **Authors** | **Year** | **Region** | **Ethnicity**  **(n)** | **Gender (n)** | **Sample size** | **Diagnostic tools (Duration and sleep data capture for EEG)** | **Age**  **at symptoms onset** | **Age**  **at diagnosis** | **Study design** | **Clinical features**  **(Number of patients with for whom clinical features was reported /Number of patients)** |
| Branch et al.^1^ | 1979 | U.S. | Not reported | Male (1) | 1 | EEG  (Neither EEG duration nor whether sleep was obtained were reported) | 6 months | Not reported | Descriptive study | Mixed (flexor/extensor) spasms (1/1) |
| Kellaway et al.^2^ | 1979 | U.S. | Not reported | Not reported | 24 | EEG  (24 hrs duration, but whether sleep was obtained was not reported) | Not reported | ​​Not reported | Descriptive study | Mixed (flexor/extensor) spasms (24/24)  Behavioral arrest (3/24)  Asymmetric/focal spasms (1/24) |
| Willis and Rosman^3^ | 1980 | U.S. | Not reported | Female (2) | 2 | EEG  (Neither EEG duration nor whether sleep was obtained were reported) | Patient 1:  Not reported  Patient 2: 6.5 months | Not reported | Descriptive study | Flexor spasms (2/2) |
| Curatolo et al.^4^ | 1983 | Italy | Not reported | Female (1) | 1 | EEG  (Neither EEG duration nor whether sleep was obtained were reported) | 4 months | Not reported | Descriptive study | Flexor spasms (1/1) |
| Hattori et al.^5^ | 1985 | Japan | Not reported | Male (1) | 1 | EEG  (Neither EEG duration nor whether sleep was obtained were reported) | 12 months | Not reported | Descriptive study | Flexor spasms (1/1) |
| Palm et al.^6^ | 1986 | Sweden | Not reported | Male (3)  Female (2) | 5^a^ | EEG  (Neither EEG duration nor whether sleep was obtained were reported) | Patient 1: 6.5 months  Patient 2: 4 months  Patient 3: 4 months  Patient 5: 4 months  Patient 6: 3.5 months | Patient 1: 9 months  Others: Not reported | Descriptive study | Flexor spasms (3/5)  Myoclonic jerks (2/5)  Asymmetric/focal spasms (1/5)  Hemi-body convulsions (1/5)  Opisthotonos (1/5) |
| Hrachovy et al.^7^ | 1987 | U.S. | Not reported | Male (1) | 1^a^ | EEG  (Neither EEG duration nor whether sleep was obtained were reported) | Patient 1: 20 months | Not reported | Descriptive study | Flexor spasms (1/1) |
| Roos et al.^8^ | 1987 | Netherlands | Not reported | Male (2) | 2 | EEG  (Neither EEG duration nor whether sleep was obtained were reported) | Patient 1: 6 months  Patient 2: 3 months | Not reported | Descriptive study | Extensor spasm (1/2)  Flexor spasms (1/2)  Myoclonic jerks (1/2) |
| Nolte et al.^9^ | 1988 | Germany | Not reported | Male (29)  Female (25) | 54 | EEG  (24-hour period, but whether sleep was obtained was not reported) | Not reported | Not reported | Descriptive study | Myoclonic jerks (24/54)  Flexor spasms (22/54)  Upward eye movement (17/54)  Mixed (flexor/extensor) spasms (16/54)  Extensor spasms (8/54) |
| Ichiba^10^ | 1990 | Japan | Not reported | Male (1)  Female (1) | 2 | EEG  (Neither EEG duration nor whether sleep was obtained were reported) | Patient 1: 4 months  Patient 2: 6 months | Patient 1: Not reported  Patient 2: 6 months | Descriptive study | Tonic spasms (2/2) |
| Donat and Wright^11^ | 1991 | U.S. | Not reported | Male (6)  Female (5) | 11 | 4 patients:  EEG (50 minutes, but whether sleep was obtained was not reported)  3 patients:  EEG (3-7 hours, but whether sleep was obtained was not reported)  4 patients:  Routine-length EEG recordings plus 24 hour remote video-EEG cable telemetry recordings, but whether sleep was obtained was not reported) | Mean: 6 months  Range: 3-9 months | Not reported | Descriptive study | Mixed (flexor/extensor) spasms (6/11)  Eye movement unspecified (4/11)  Facial movements/grimace (4/11)  Asymmetric spasms (2/11)  Upward eye movement (2/11)  Extensor spasms (1/11)  Ocular adduction (1/11) |
| Feng et al.^12^ | 1991 | China | Not reported | Male (67)  Female (38) | 105 | EEG  (Neither EEG duration nor whether sleep was obtained were reported) | Range: 1-36 months | Not reported | Descriptive study | Flexor spasms (74/105)  Head nodding (74/105)  Extensor spasms (13/105)  Mixed (flexor/extensor) spasms (7/105)  Frightening expression (3/105)  Spasms of the corner of the mouth (1/105) |
| Carrazana et al. ^13^ | 1993 | U.S. | Not reported | Male (7)  Female (9) | 16 | EEG  (Neither EEG duration nor whether sleep was obtained were reported)  Case 3:  EEG (Prolonged EEG, but whether sleep was obtained was not reported) | Mean: 3 months  Range: 1 -8 months | Not reported | Descriptive study | Flexor spasms (13/16)  Mixed (flexor/extensor) spasms (2/16)  Extensor spasms (1/16) |
| Cusmai et al. ^14^ | 1993 | U.S. | Not reported | Not reported | 24^a^ | EEG  (Neither EEG duration nor whether sleep was obtained were reported) | Porencephalic lesions group:  Mean: 6.3 months  Range: 5-9 months  Diffuse cerebral lesions group:  Mean: 5.1 months  Range: 4-8 months | Not reported | Descriptive study | Asymmetric spasms (17/24)  Symmetric spasms (7/24) |
| Fukumizu et al. ^15^ | 1993 | Japan | Not reported | Male (1)  Female (2) | 3 | EEG  (Neither EEG duration nor whether sleep was obtained were reported) | Patient 1: 3 months  Patient 2: 2 months  Patient 3: 3 months | Patient 1: 3 months  Patient 2: Not reported  Patient 3: Not reported | Descriptive study | Flexor spasms (3/3)  Postural spasms (2/3)  Symmetric spasms (1/3) |
| Fusco and Vigevano^16^ | 1993 | Italy | Not reported | Not reported | 36 | EEG  (Neither EEG duration nor whether sleep was obtained were reported) | Range: 2-11 months | Not reported | Descriptive study | Flexor spasms (18/36)  Extensor spasms (10/36)  Asymmetric spasms (8/36)  Mixed (flexor/extensor) spasms (8/36) |
| Koo et al.^17^ | 1993 | Canada | Not reported | Male (33)  Female (24) | 57 | EEG  (Neither EEG duration nor whether sleep was obtained were reported) | Range: 4 weeks -13 months | Not reported | Descriptive study | Flexor spasms (46/57)  Mixed (flexor/extensor) spasms (8/57)  Extensor spasms (3/57) |
| Vigevano et al.^18^ | 1993 | Italy | Not reported | Male (12)  Female (19) | 31 | EEG  (Prolonged wakefulness and sleep) | Mean:  5 months 14 days  Range:  4 months to 7 months | Not reported | Descriptive study | Mixed (flexor/extensor) spasms (31/31) |
| du Plessis et al. ^19^ | 1994 | U.S. | Not reported | Male (1)  Female (3) | 4 | EEG  (Neither EEG duration nor whether sleep was obtained were reported) | Range: 6-7 months | Not reported | Descriptive study | Extensor spasms (1/4)  Flexor spasms (1/4)  Symmetric spasms (1/4)  Tonic upgaze (1/4) |
| Fois et al.^20^ | 1994 | Italy | Not reported | Male (1) | 1 ^a^ | EEG  (Neither EEG duration nor whether sleep was obtained were reported) | 5 months | ​​Not reported | Descriptive study | Flexor spasms (1/1) |
| ​​Luovigsson et al.^21^ | 1994 | Iceland | Not reported | Male (10)  Female (3) | 13 | EEG  (Neither EEG duration nor whether sleep was obtained were reported) | Mean: 5.6 months  Range: 2-11 months | Not reported | Descriptive study | Flexor spasms (13/13) |
| Menezes et al.^22^ | 1994 | Canada | Not reported | Female (10) | 10^a^ | EEG  (Neither EEG duration nor whether sleep was obtained were reported) | 10 out of 14 patients developed before 3 months | Not reported | Descriptive study | Flexor spasms (8/10)  Extensor spasms (1/10)  Mixed (flexor/extensor) spasms (1/10) |
| Asanuma et al.^23^ | 1995 | Japan | Not reported | Male (2) | 2 | ​​EEG  (Neither EEG duration nor whether sleep was obtained were reported) | 5 months | ​​Not reported | Descriptive study | Tonic spasms (2/2)  Head nodding (1/2) |
| Haga et al.^24^ | 1995 | Japan | Not reported | Male (24)  Female (18) | 42 | EEG  (Neither EEG duration nor whether sleep was obtained were reported) | Range: 2-11 months | Not reported | Descriptive study | Flexor spasm (28/42)  Mixed (flexor/extensor) spasms (9/42)  Asymmetric spasms (6/42)  Eye deviation (6/42)  Extensor spasm (5/42) |
| Bingham et al.^25^ | 1996 | U.S. | White (1) | Female (1) | 1 | EEG  (Neither EEG duration nor whether sleep was obtained were reported) | 6.5 months | 7 months | Descriptive study | Flexor spasms. (1/1) |
| Dakshinamurtyet.al.^26^ | 1996 | U.S. | Not reported | Male (21)  Female (8) | 29 | EEG  (Neither EEG duration nor whether sleep was obtained were reported) | Mean: 8.8 ± 6.7 months  Range: 1-24 months | Not reported | Descriptive study | Flexor spasms (27/29)  Mixed (flexor/extensor) spasms (2/29) |
| Kamei et al. ^27^ | 1996 | Japan | Asian (1) | Male (1) | 1 | EEG  (Neither EEG duration nor whether sleep was obtained were reported) | 2 months old | Not reported | Descriptive study | Asymmetric spasms (1/1)  Extensor spasms (1/1)  Flexor spasms (1/1)  Symmetric spasms (1/1) |
| Sharma et al.^28^ | 1996 | India | Not reported | Male (16)  Female (9) | 25 | EEG  (Neither EEG duration nor whether sleep was obtained were reported) | Mean: 5.2 months  Range: l-10 months | Not reported | Descriptive study | Symmetric spasms (25/25)  Flexor spasms (19/25)  Mixed (flexor/extensor) spasms (4/25)  Extensor spasms (2/25) |
| Acharya et al.^29^ | 1997 | U.S. | Not reported | Male (2) | 2 ^a^ | EEG  (Prolonged EEG  Range: 1 to 6 days  Mean: 3.5 days; but whether sleep was obtained was not reported) | Mean: 5 months  Range: 1 day to 14 months | Not reported | Descriptive study | Mixed (flexor/extensor) spasms (2/2)  Symmetric spasms (2/2) |
| Mitsudome et al.^30^ | 1997 | Japan | Not reported | Male (1) | 1 | EEG  (Duration: not reported; waking and sleeping EEG) | 6 months | 7 months | Descriptive study | Tonic spasms (1/1) |
| Kubota et al.^31^ | 1999 | Japan | Not reported | Male (1)  Female (7) | 8 ^a^ | EEG  (Neither EEG duration nor whether sleep was obtained were reported) | Range:  2 days - 9 months  Mean: 3.4 months | Not reported | Descriptive study | Asymmetric spasms (4/8)  Symmetric spasms (4/8) |
| Castano et al.^32^ | 2000 | Canada | Not reported | Male (2) | 2 ^a^ | EEG  (Neither EEG duration nor whether sleep was obtained were reported) | Not reported | Not reported | Descriptive study | Eyes deviation (1/2)  Flexor spasms (1/2)  Head nodding (1/2)  Myoclonic jerks (1/2) |
| Kishi et al.^33^ | 2000 | Japan | Asian (1) | Female (1) | 1 ^a^ | EEG  (Duration: Not reported; EEG during sleep) | 5 months | 5 months | Descriptive study | Tonic spasms (1/1) |
| Pineda et al.^34^ | 2000 | Spain | Not reported | Female (1) | 1 | EEG  (Long term during sleep cycle) | 7 months | Not reported | Descriptive study | Flexor spasms (1/1) |
| Sfaello et al.^35^ | 2000 | France | Not reported | Male (1) | 1 ^a^ | EEG  (Neither EEG duration nor whether sleep was obtained were reported) | 5 months | Not reported | Descriptive study | Myoclonic jerks (1/1) |
| Gaily et al.^36^ | 2001 | Finland | Not reported | Male (27)  Female (17) | 44 | EEG  (3-8 hours, but whether sleep was obtained was not reported) | Mean: 5.3 months  Range 0.9 - 9 months | Not reported | Descriptive study | Symmetric spasms (34/44)  Subtle spasms (7/44)  Asymmetric spasms (3/44) |
| Hwang^37^ | 2001 | South Korea | Asian (358) | Male (219)  Female (139) | 358 | EEG  (Neither EEG duration nor whether sleep was obtained were reported) | Not reported | Not reported | Descriptive study | Flexor spasms (212/358)  Mixed (flexor/extensor) spasms (61/358)  Extensor spasms (60/358)  Atypical spasms (17/358)  Akinetic spasms (8/358) |
| Suastegui et al.^38^ | 2001 | Mexico | Latino (72) | Male (22)  Female (50) | 72 | EEG  (Neither EEG duration nor whether sleep was obtained were reported) | Mean: 9 months | Not reported | Descriptive study | Flexor spasms (51/72)  Extensor spasms (11/72)  Mixed (flexor/extensor) spasms (10/72) |
| Sugai et al.^39^ | 2001 | Japan | Asian (30) | Male (18)  Female (12) | 30^a^ | EEG  (Neither EEG duration nor whether sleep was obtained were reported) | Range: 3 -12 months | Not reported | Descriptive study | Flexor spasms (20/30)  Extensor spasms (10/30) |
| Wong^40^ | 2001 | Hong Kong | Not reported | Not reported | 82^a^ | EEG  (Neither EEG duration nor whether sleep was obtained were reported) | Range: 0-3 months | Not reported | Descriptive study | Flexor spasms (70/82)  Mixed (flexor/extensor) spasms (6/82)  Extensor spasms (5/82)  Myoclonic jerks (1/82) |
| Zhou et al.^41^ | 2001 | China | Not reported | Male (41)  Female (21) | 62 | EEG  (Neither EEG duration nor whether sleep was obtained were reported) | Range: 4 - 18 months | Not reported | Descriptive study | Flexor spasms (30/62)  Extensor spasms (15/62)  Mixed (flexor/extensor) spasms (13/62)  Atypical spasms (4/62) |
| De Menezes and Rho^42^ | 2002 | U.S. | Not reported | Male (18)  Female (8) | 26 | EEG  (Neither EEG duration nor whether sleep was obtained were reported) | Mean: 5.8 months  Range: 1–18 months | Not reported | Descriptive study | Mixed (flexor/extensor) spasms (13/26)  Asymmetric spasms (6/26)  Flexor spasms (6/26)  Eye deviation (5/26)  Head deviation (5/26)  Upward eye movement (5/26) |
| Gudino et al.^43^ | 2002 | Spain | Not reported | Male (1) | 1 | EEG  (Neither EEG duration nor whether sleep was obtained were reported) | 5 months | Not reported | Descriptive study | Extensor spasms (1/1) |
| Lortie et al.^44^ | 2002 | Canada and France | Not reported | Not reported | 11^a^ | EEG  (Neither EEG duration nor whether sleep was obtained were reported) | Before 1 year old | Not reported | Descriptive study | Asymmetric spasms (9/11)  Nystagmoid eye movements (2/11)  Head deviation (1/11) |
| Topcu et al.^45^ | 2002 | Turkey | Not reported | Male (1) | 1 | EEG  (Neither EEG duration nor whether sleep was obtained were reported) | Not reported | Not reported | Descriptive study | Myoclonic jerks (1/1) |
| Yamatogi and Ohtahara^46^ | 2002 | Japan | Not reported | Male (9)  Female (7) | 16 | EEG  (Long term during sleep cycle) | All patients’ onset age within 3 months (12 patients had their onset within 1 month) | Not reported | Descriptive study | Tonic spasms (16/16) |
| Golomb, et al.^47^ | 2004 | U.S. | Not reported | Male (1) | 1 | EEG  (Neither EEG duration nor whether sleep was obtained were reported) | 6 months | Not reported | Descriptive study | Myoclonic jerks (1/1)  Head nodding (1/1) |
| Singhi & Ray^48^ | 2005 | India | Asian (165) | Male (112)  Female (53) | 165 | EEG  (Neither EEG duration nor whether sleep was obtained were reported) | Mean: 6.1±3.4 months  Range: 1 month -1 year 7 months | Not reported | Descriptive study | Flexor spasms (122/165)  Mixed (flexor/extensor) spasms (28/165)  Extensor spasms (10/165)  Unclear spasms (5/165) |
| Tsao et al.^49^ | 2005 | U.S. | White (1) | Female (1) | 1 | EEG  (Neither EEG duration nor whether sleep was obtained were reported) | 5 months | Not reported | Descriptive study | Flexor spasms (1/1) |
| Bahi-Buisson et al.^50^ | 2006 | France | Not reported | Male (11) | 11^a^ | EEG  (>2 hours, but whether sleep was obtained was not reported) | Mean: 9.5 months  Median: 10 months | Not reported | Descriptive study | Extensor spasms (5/11)  Mixed (flexor/extensor) spasms (5/11)  Flexor spasms (1/11) |
| Kang et al.^51^ | 2006 | South Korea | Not reported | Male (13)  Female (14) | 27 | EEG  (Neither EEG duration nor whether sleep was obtained were reported) | 25 patients: <12 months  2 patients: > 12 months | Not reported | Descriptive study | Asymmetric spasms (22/27)  Symmetric spasms (5/27) |
| Kwon et al.^52^ | 2006 | South Korea | Not reported | Male (17)  Female (3) | 20 | EEG  (Neither EEG duration nor whether sleep was obtained were reported) | Median: 6.5 months  Range: 3- 24 months | Not reported | Descriptive study | Flexor spasms (11/20)  Extensor spasms (8/20)  Mixed (flexor/extensor) spasms (1/20) |
| Lee et al.^53^ | 2006 | South Korea | Not reported | Male (1) | 1 | EEG  (Neither EEG duration nor whether sleep was obtained were reported) | Not reported | Not reported | Descriptive study | Flexor spasms (1/1)  Vocalization (1/1) |
| Soman et al.^54^ | 2006 | Canada | Not reported | Male (4) | 4 | EEG  (Neither EEG duration nor whether sleep was obtained were reported) | Patient 1: 9 months  Patient 2: 7 months  Patient 3: 11 months  Patient 4: 10 months | Patient 4: 10 months  Other patients: Not reported | Descriptive study | Extensor spasms (2/4)  Acoustic hypersensitivity/startle (1/4)  Associated crying (1/4)  Asymmetric spasms (1/4)  Eye fluttering/blinking (1/4)  Flexor spasms (1/4)  Head nodding (1/4)  Upward eye movement (1/4) |
| Erol et al.^55^ | 2007 | Turkey | Not reported | Female (1) | 1 | EEG  (Neither EEG duration nor whether sleep was obtained were reported) | 9 months | Not reported | Descriptive study | Flexor spasms (1/1) |
| Ito et al.^56^ | 2007 | Japan | Not reported | Female (1) | 1 | EEG  (Neither EEG duration nor whether sleep was obtained were reported) | Not reported | Not reported | Descriptive study | Tonic spasms (1/1) |
| Lapatsanis and Lapatsanis^57^ | 2007 | Greece | Not reported | Male (1) | 1^a^ | EEG  (Neither EEG duration nor whether sleep was obtained were reported) | Not reported | Not reported | Descriptive study | Behavioral arrest (1/1)  Eye jerk (1/1) |
| Mikati et al.^58^ | 2007 | Lebanon | Not reported | Female (1) | 1 | EEG  (Neither EEG duration nor whether sleep was obtained were reported) | 4 months | Not reported | Descriptive study | Flexor spasms (1/1) |
| Yamamoto et al.^59^ | 2007 | Japan | Not reported | Female (1) | 1^a^ | EEG  (Neither EEG duration nor whether sleep was obtained were reported) | Not reported | 3-6 months | Descriptive study | Head nodding (1/1) |
| Imataka et al.^60^ | 2007 | Japan | Not reported | Male (1) | 1 | EEG  (Neither EEG duration nor whether sleep was obtained were reported) | 5 months | Not reported | Descriptive study | Tonic spasms (1/1)  Flexor spasms (1/1) |
| Kuzmanić-Šamija et al.^61^ | 2008 | Croatia | Not reported | Male (22)  Female (15) | 37 | EEG  (Neither EEG duration nor whether sleep was obtained were reported) | Median: 7 months  Range: 1–11 months | Not reported | Descriptive study | Flexor spasms (24/37)  Mixed (flexor/extensor) spasms (10/37)  Extensor spasms (3/37) |
| Poirier et al.^62^ | 2008 | France | Not reported | Male (1) | 1 | EEG  (Neither EEG duration nor whether sleep was obtained were reported) | 2 months | 4 months | Descriptive study | Upward eye movement (1/1) |
| Wallerstein et al.^63^ | 2008 | U.S. | Not reported | Female (1) | 1^a^ | EEG  (Neither EEG duration nor whether sleep was obtained were reported) | 4 months | Not reported | Descriptive study | Myoclonic jerks (1/1)  Vocalization (1/1) |
| Ruggieri et al.^64^ | 2009 | Italy | Not reported | Male (4)  Female (6) | 10 | EEG  (Neither EEG duration nor whether sleep was obtained were reported) | Range: 3-9 months old | Not reported | Descriptive study | Symmetric spasms (6/10)  Mixed (flexor/extensor) spasms (5/10)  Asymmetric spasms (4/10)  Flexor spasm (2/10)  Head nodding (1/10) |
| Ibrahim et al.^65^ | 2010 | Pakistan | Not reported | Not reported | 44^a^ | EEG  (Neither EEG duration nor whether sleep was obtained were reported) | Mean: 5 ± 1.4 months | Mean: 6.5 ± 2.3 months | Descriptive study | Flexor spasms (23/44)  Mixed (flexor/extensor) spasms (15/44)  Extensor spasms (6/44) |
| Kumaran et al.^66^ | 2010 | U.K. | Not reported | Male (2)  Female (2) | 4^a^ | EEG  (Neither EEG duration nor whether sleep was obtained were reported) | Patient 1: 9 months  Patient 2: 3 months  Patient 3: 9 months  Patient 5: 4.5 months | Not reported | Descriptive study | Flexor spasms (2/4)  Extensor spasms (1/4)  Head nodding (1/4)  Myoclonic jerks (1/4) |
| Marashly et al.^67^ | 2010 | U.S. | Not reported | Male (1) | 1 | EEG  (Neither EEG duration nor whether sleep was obtained were reported) | Not reported | Not reported | Descriptive study | Associated crying (1/1)  Extensor spasms (1/1)  Head nodding (1/1)  Symmetric spasms (1/1)  Upward eye movement (1/1) |
| Caraballo et al.^68^ | 2011 | Argentina, Uruguay, Italy | Not reported | Male (9)  Female (7) | 16 | EEG  (12-24 hours, but whether sleep was obtained was not reported) | Mean: 9 months  Median: 7 months  Range: 4 - 30 months. | Not reported | Descriptive study | Flexor spasms (10/16)  Asymmetric spasms (9/16)  Mixed (flexor/extensor) spasms (6/16) |
| Fujita et al.^69^ | 2011 | Japan | Not reported | Male (2)  Female (2) | 4^a^ | EEG  (Neither EEG duration nor whether sleep was obtained were reported) | Patient 2: 8 months  Patient 4: 10 months  Patient 6: 6 months  Patient 7: 3 months | Not reported | Descriptive study | Tonic spasms (4/4)  Flexor spasms (1/4) |
| Khreisat^70^ | 2011 | Jordan | Not reported | Male (37)  Female (13) | 50 | EEG  (Neither EEG duration nor whether sleep was obtained were reported) | Mean 4.8 months  Range: 1 month to 1 year and 6 months | Not reported | Descriptive study | Flexor spasms (21/50)  Mixed (flexor/extensor) spasms (18/50)  Extensor spasms (11/50)  Asymmetric spasms (5/50) |
| Lujic et al.^71^ | 2011 | Croatia | Not reported | Male (9)  Female (2) | 11 | EEG  (Neither EEG duration nor whether sleep was obtained were reported) | Range: 5- 16 months | Not reported | Descriptive study | Flexor spasms (10/11)  subtle spasms (1/11) |
| Melani et al.^72^ | 2011 | Italy | Not reported | Male (1)  Female (1) | 2^a^ | EEG  (prolonged/long term; but whether sleep was obtained was not reported) | Patient 1: 5 months  Patient 6: 2 months | Not reported | Descriptive study | Flexor spasms (2/2)  Eyelid jerks (1/2) |
| Mizuno et al.^73^ | 2011 | Japan | Not reported | Female (1) | 1 | EEG  (Neither EEG duration nor whether sleep was obtained were reported) | 5 months | 5 months | Descriptive study | Mixed (flexor/extensor) spasms (1/1) |
| Mordekar et al.^74^ | 2011 | U.K. | White (4) | Male (3)  Female (1) | 4 | EEG  (Neither EEG duration nor whether sleep was obtained were reported) | Patient 1: 4 months  Patient 2: 6 months  Patient 3: 6 months  Patient 4: 5 months | Not reported | Descriptive study | Eye deviation to one side (2/4)  Mixed (flexor/extensor) spasms (3/4)  Asymmetric spasms (1/4)  Head deviation (1/4) |
| Saito et al.^75^ | 2011 | Japan | Not reported | Male (1) | 1 | EEG  (Neither EEG duration nor whether sleep was obtained were reported) | 3 months | Not reported | Descriptive study | Asymmetric spasms (1/1)  Eye deviation to one side (1/1)  Myoclonic jerks (1/1) |
| Taddio et al.^76^ | 2011 | Italy | Not reported | Male (1)  Not reported (2) | 3 | EEG  (Neither EEG duration nor whether sleep was obtained were reported) | Not reported | Not reported | Descriptive study | Flexor spasms (1/3)  Mixed (flexor/extensor) spasms (1/3)  Extensor spasms (1/3)  Tonic spasms (2/3)  Symmetric spasms (2/3)  Swallowing and vomiting (1/3)  Associated crying (3/3) |
| Auvin et al.^77^ | 2012 | France | Not reported | Male (44)  Female (39) | 83 | EEG  (Neither EEG duration nor whether sleep was obtained were reported) | Not reported | Median: 6 months | Descriptive study | Symmetric spasms (53/83)  Flexor spasms (46/83)  Asymmetric spasms (30/83)  Mixed (flexor/extensor) spasms (24/83)  Extensor spasms (13/83) |
| Malik et al.^78^ | 2012 | Pakistan | Not reported | Not reported | 328 ^a^ | EEG  (Neither EEG duration nor whether sleep was obtained were reported) | Mean: 3.5±2.8 months  Range: 1-24 months | Not reported | Descriptive study | Mixed (flexor/extensor) spasms (184/328)  Flexor spasms (79/328)  Extensor spasms (39/328)  Asymmetric spasms (26/328) |
| Richard-Mousnier et al.^79^ | 2012 | France | Not reported | Female (1) | 1 | EEG  (Duration: Not reported; EEG during sleep) | 2 years old | Not reported | Descriptive study | Head nodding (1/1)  Tonic spasms (1/1) |
| Singh et al.^80^ | 2012 | India | Not reported | Female (1) | 1 | EEG  (Neither EEG duration nor whether sleep was obtained were reported) | Not reported | Not reported | Descriptive study | Flexor spasms (1/1) |
| Writzl et al.^81^ | 2012 | Slovenia | Not reported | Female (1) | 1 | EEG  (Neither EEG duration nor whether sleep was obtained were reported) | 3.5 months | Not reported | Descriptive study | Flexor spasms (1/1) |
| Bayram et al.^82^ | 2013 | Turkey | White (2) | Female (2) | 2 | EEG  (Neither EEG duration nor whether sleep was obtained were reported) | Patient 1: 3 months  Patient 2: 3 months | Not reported | Descriptive study | Flexor spasms (2/2)  Focal eye signs (1/2) |
| Kaushik et al.^83^ | 2013 | India | Not reported | Male (120)  Female (28) | 148 | EEG  (Neither EEG duration nor whether sleep was obtained were reported) | Mean: 5.3 months | Mean: 13.1 months | Descriptive study | Flexor spasms (113/148)  Mixed (flexor/extensor) spasms (26/148)  Extensor spasms (9/148) |
| Lacaze et al.^84^ | 2013 | France | Not reported | Male (1) | 1 | EEG  (Neither EEG duration nor whether sleep was obtained were reported) | 5 months | 6 months | Descriptive study | Eye deviation to one side (R) (1/1)  Flexor spasms (1/1)  Mixed (flexor/extensor) spasms (1/1)  Upward eye movement (1/1) |
| Mangano et al.^85^ | 2013 | Italy | Not reported | Male (1) | 1 | EEG  (Neither EEG duration nor whether sleep was obtained were reported) | 8 months | Not reported | Descriptive study | Symmetric spasms (1/1) |
| Alrifai et al.^86^ | 2014 | Saudi Arabia | Not reported | Not reported | 68^a^ | EEG  (Neither EEG duration nor whether sleep was obtained were reported) | HNMDs group median: 4.75 months  Other etiologies group median:5 months | HNMDs group  Median: 7.5 months  Other etiologies group:  Not reported | Descriptive study | Flexor spasms (42/68)  Subtle spasms (16/68)  Extensor spasms (10/68) |
| Çelik et al.^87^ | 2014 | Turkey | Not reported | Female (1) | 1 | EEG  (Neither EEG duration nor whether sleep was obtained were reported) | 4~5 months | Not reported | Descriptive study | Flexor spasms (1/1) |
| Holder et al.^88^ | 2014 | U.S. | Not reported | Male (1) | 1 | EEG  (Neither EEG duration nor whether sleep was obtained were reported) | 15 months | Not reported | Descriptive study | Tonic spasms (1/1) |
| Pontrelli et al.^89^ | 2014 | Italy | Not reported | Male (1) | 1 | EEG  (Neither EEG duration nor whether sleep was obtained were reported) | 4 months | Not reported | Descriptive study | Extensor spasms (1/1)  Flexor spasms (1/1) |
| Sukhudyan et al.^90^ | 2014 | Armenia, Sofia Bulgaria, Italy | Not reported | Male (1)  Female (2) | 3 ^a^ | EEG  (Neither EEG duration nor whether sleep was obtained were reported) | Patient 2: 4 months  Patient 6: 5 months  Patient 10: 4 months | Not reported | Descriptive study | Flexor spasms (2/3)  Asymmetric spasms (1/3)  Eye deviation to one side R (1/3)  Head deviation (1/3) |
| Vega et al.^91^ | 2014 | U.K. | Not reported | Female (1) | 1 | EEG  (Neither EEG duration nor whether sleep was obtained were reported) | 9 months | Not reported | Descriptive study | Flexor spasms (1/1) |
| Ciccone et al.^92^ | 2015 | Zambia | Not reported | Male (1) | 1 | EEG  (Duration: Not reported; EEG during wake and sleep) | 6 months | Not reported | Descriptive study | Flexor spasms (1/1) |
| Gulati et al.^93^ | 2015 | India | Not reported | Male (234)  Female (76) | 310 | EEG  (Duration: Not reported; EEG during sleep) | Median: 5 months  Range: 2.5-7 months | Not reported | Descriptive study | Flexor spasms (243/310)  Mixed (flexor/extensor) spasms (35/310)  Extensor spasms (32/310) |
| Güveli et al.^94^ | 2015 | Turkey | Not reported | Male (64)  Female (45) | 109 | EEG  (Neither EEG duration nor whether sleep was obtained were reported) | Mean: 6 months | Not reported | Descriptive study | Symmetric spasms (104/109)  Asymmetric spasms (5/109) |
| Papetti et al.^95^ | 2015 | Italy | Mixed race (1) | Male (1) | 1 | Interictal EEG  (Neither EEG duration nor whether sleep was obtained were reported) | 3 months | Not reported | Descriptive study | Symmetric spasms (1/1) |
| Xixis and Mikati^96^ | 2015 | U.S. | Not reported | Female (1) | 1 | EEG  (Neither EEG duration nor whether sleep was obtained were reported) | 11 months | Not reported | Descriptive study | Myoclonic jerks (1/1)  Tonic spasms (1/1) |
| Arican et al.^97^ | 2016 | Turkey | Not reported | Male (1) | 1 | EEG  (Neither EEG duration nor whether sleep was obtained were reported) | Not reported | Not reported | Descriptive study | Flexor spasms (1/1)  Head nodding (1/1)  Myoclonic jerks (1/1) |
| Caraballo et al. ^98^ | 2016 | Argentina | Not reported | Male (29)  Female (19) | 48 | EEG  (12-24 hours, but whether sleep was obtained was not reported) | Median: 10 months  Mean: 23 months | Not reported | Descriptive study | Asymmetric spasms (24/48)  Flexor spasms (23/48)  Mixed (flexor/extensor) spasms (14/48)  Extensor spasms (11/48) |
| Caraballo  et al.^99^ | 2016 | Argentina | Not reported | Male (8)  Female (4) | 12 | EEG  (Neither EEG duration nor whether sleep was obtained were reported) | Mean: 4.5 months  Median: 6 months  Range: 3.5-7 months | Not reported | Descriptive study | Flexor spasms (6/12)  Mixed (flexor/extensor) spasms (5/12)  Extensor spasms (1/12) |
| Lee and Hur^100^ | 2016 | South Korea | Not reported | Female (1) | 1 | EEG  (Neither EEG duration nor whether sleep was obtained were reported) | 6 months | Not reported | Descriptive study | Flexor spasm (1/1) |
| Raviglione et al.^101^ | 2016 | Italy | Not reported | Male (1) | 1 | EEG  (Neither EEG duration nor whether sleep was obtained were reported) | 3 months | Not reported | Descriptive study | Flexor spasms (1/1)  Upward eye deviation (1/1) |
| Yilmaz et al.^102^ | 2016 | Turkey | Not reported | Male (117)  Female (99) | 216 | EEG  (Neither EEG duration nor whether sleep was obtained were reported) | Median: 7 months | Not reported | Descriptive study | Flexor spasm (102/216)  Mixed (flexor/extensor) spasms (67/216)  Extensor spasm (45/216)  Subtle spasm (2/216) |
| Benitez et al.^103^ | 2017 | U.S. | Not reported | Not reported | 8^a^ | EEG  (Neither EEG duration nor whether sleep was obtained were reported)) | Median: 1 year  Range: 0.4-2.7 years | Not reported | Descriptive study | Asymmetric spasms (5/8)  Extensor spasm (5/8)  Head nodding (3/8)  Abnormal eye movement (2/8)  Flexor spasm (2/8)  Myoclonic jerk (2/8)  Facial movements/grimace (1/8) |
| Caraballo et al.^104^ | 2017 | Argentina & Italy | Not reported | Male (9)  Female (7) | 16 | EEG  (12-24 hours, but whether sleep was obtained was not reported) | Mean: 11 months  Range: 2 - 84 months | Not reported | Descriptive study | Flexor spasms (8/16)  Mixed (flexor/extensor) spasms (6/16)  Extensor spasms (2/16) |
| Gul Mert et al.^105^ | 2017 | Turkey | Not reported | Male (41)  Female (63) | 104 | EEG  (Neither EEG duration nor whether sleep was obtained were reported) | Mean: 5 ± 2.78 months | Range 1-15 months | Descriptive study | Mixed (flexor/extensor) spasms (45/104)  Flexor spasm (37/104).  Extensor spasms (22/104) |
| Kasinathan et al.^106^ | 2017 | India | Not reported | Female (1) | 1 | EEG  (Neither EEG duration nor whether sleep was obtained were reported) | 8 months | Not reported | Descriptive study | Asymmetric spasms (1/1) |
| Kobayashi et al.^107^ | 2017 | Japan | Not reported | Female (1) | 1 | EEG  (Long term, but whether sleep was obtained was not reported) | 1 year | Not reported | Descriptive study | Symmetric spasms (1/1) |
| Kvernadze et al.^108^ | 2017 | Georgia | Not reported | Male (17)  Female (14) | 31 | EEG  (Long term during sleep) | Mean: 6.3 months  Range: 3- 17 months | Not reported | Descriptive study | Symmetric spasms (25/31)  Asymmetric spasms (6/31) |
| Jiang et al.^109^ | 2017 | China | Not reported | Female (1) | 1 | EEG  (Neither EEG duration nor whether sleep was obtained were reported) | Not reported | Not reported | Descriptive study | Head nodding (1/1)  Myoclonic jerks (1/1) |
| Pereira et al.^110^ | 2017 | France | Not reported | Male (1)  Female (1) | 2^a^ | EEG  (Neither EEG duration nor whether sleep was obtained were reported) | Patient 1: 4 months  Patient 2: 4 months | Not reported | Descriptive study | Flexor spasms (2/2)  Asymmetric spasms (1/2)  Symmetric spasms (1/2) |
| Ciara et al.^111^ | 2018 | Poland | White (5)  Asian (2) | Male (2)  Female (1) | 3 | EEG  (Neither EEG duration nor whether sleep was obtained were reported) | Patient 1: 4 months  Patient 2: 5 months  Patient 3: Not reported | Patient 3: 6 months  Other patients: Not reported | Descriptive study | Myoclonic jerks (3/3)  Acoustic hypersensitivity (2/3) |
| Kulsoom et al.^112^ | 2018 | Pakistan | Not reported | Male (24)  Female (12) | 36 | EEG  (Neither EEG duration nor whether sleep was obtained were reported) | Mean: 4.6±2.0 months | Mean: ​​6.5±3.5 months | Descriptive study | Myoclonic jerks (14/36)  Flexor spasms (11/36)  Mixed (flexor/extensor) spasms (10/36)  Extensor spasms (1/36) |
| Ben Abdelaziz et al.^113^ | 2018 | Tunisia | Not reported | Male (21)  Female (17) | 38 | EEG  (Neither EEG duration nor whether sleep was obtained were reported) | Median: 5 months  Range: 1-20 months | Not reported | Descriptive study | Flexor spasms (31/38)  Mixed (flexor/extensor) spasms (5/38)  Extensor spasms (2/38) |
| Chong et al.^114^ | 2019 | Japan | Not reported | Male (1) | 1 | EEG  (Neither EEG duration nor whether sleep was obtained were reported) | 7 months. | ​​Not reported | Descriptive study | Extensor spasms (1/1)  Head nodding (1/1)  Upward eye deviation (1/1) |
| Li et al.^115^ | 2019 | China | Asian (1) | Male (1) | 1 | EEG  (Neither EEG duration nor whether sleep was obtained were reported) | 6 months | Not reported | Descriptive study | Flexor spasms (1/1) |
| Yoshida et al.^116^ | 2019 | Japan | Not reported | Female (1) | 1 | EEG  (Neither EEG duration nor whether sleep was obtained were reported) | 5 months | Not reported | Descriptive study | Flexor spasms (1/1) |
| Barbarrosa et al.^117^ | 2020 | Cuba | Not reported | Male (22)  Female (17) | 39 | EEG  (Neither EEG duration nor whether sleep was obtained were reported) | 12 patients: < 4 months  23 patients: 4-8 months  4 patients: > 8 months | Not reported | Descriptive study | Symmetric spasm (36/39)  Flexor spasm (20/39)  Mixed (flexor/extensor) spasms (14/39)  Extensor spasm (5/39)  Asymmetric spasm (3/39) |
| Charkhand et al.^118^ | 2020 | Canada | White (1) | Female (1) | 1 | EEG  (Neither EEG duration nor whether sleep was obtained were reported) | 7 months | Not reported | Descriptive study | Flexor spasms (1/1) |
| Desprairies et al.^119^ | 2020 | Egyptian (1) and unknown (2) | Not reported | Male (2) | 2^a^ | EEG  (Neither EEG duration nor whether sleep was obtained were reported) | Patient 1: 5 months  Patient 3: 3 months | Not reported | Descriptive study | Extensor spasms (1/2)  Flexor spasms (1/2)  Symmetric spasms (1/2) |
| McFarlane et al.^120^ | 2020 | Canada | Not reported | Male (176)  Female (136) | 312 | EEG  (Neither EEG duration nor whether sleep was obtained were reported) | Not reported | Not reported | Descriptive study | Mixed (flexor/extensor) spasms (312/312) |
| Romano et al.^121^ | 2020 | Italy | Not reported | Female (1) | 1 | EEG  (Long term EEG during sleep) | 6 months | Not reported | Descriptive study | Flexor spasms (1/1) |
| Sharawat and Panda^122^ | 2020 | India | Not reported | Female (1) | 1 | EEG  (Neither EEG duration nor whether sleep was obtained were reported) | Not reported | Not reported | Descriptive study | Flexor spasms (1/1) |
| Zhang et al.^123^ | 2020 | China | Asian (1) | Male (1) | 1 | EEG  (Neither EEG duration nor whether sleep was obtained were reported) | 2 months | Not reported | Descriptive study | Flexor spasms (1/1) |
| Fukuoka et al.^124^ | 2021 | Japan | Not reported | Female (1) | 1 | EEG  (Neither EEG duration nor whether sleep was obtained were reported) | 7 months | Not reported | Descriptive study | Extensor spasms (1/1) |
| Sun et al.^125^ | 2021 | China | Not reported | Male (1) | 1^a^ | EEG  (Neither EEG duration nor whether sleep was obtained were reported) | Not reported | Not reported | Descriptive study | Extensor Spasms (1/1)  Head nodding (1/1) |
| Crippa et al.^126^ | 2022 | Brazil | Not reported | Male (3)  Female (1) | 4 | EEG  (Neither EEG duration nor whether sleep was obtained were reported) | Patient 1: Not reported  Patient 2: 3 months  Patient 3: Not reported  Patient 4: Not reported | Patient 1: 10 months  Patient 2: 8 months  Patient 3: 5 months  Patient 4: 4 months | Descriptive study | Tonic spasms (1/4)  Mixed (flexor/extensor) spasms (2/4)  Asymmetric spasms (1/4) |
| Haneder et al.^127^ | 2022 | Austria | Not reported | Male (9)  Female (9) | 18 | EEG  (24-72 hrs, but whether sleep was obtained was not reported) | Mean: 6.8 months  Range: 3-14 months | Not reported | Descriptive study | Asymmetric spasms (17/18)  Tonic spasms (14/18)  Abnormal eye movement (14/18)  Spasms of the corner of the mouth (2/18) |
| Sabapathy et al.^128^ | 2022 | U.S. | Not reported | Male (1) | 1 | EEG  (Neither EEG duration nor whether sleep was obtained were reported) | 7 months | Not reported | Descriptive study | Behavioral arrest (1/1)  Symmetric spasms (1/1)  Tonic spasms (1/1) |
| Madan and Chaudhuri^129^ | 2022 | India | Not reported | Female (1) | 1 | EEG  (Neither EEG duration nor whether sleep was obtained were reported) | 7 months | Not reported | Descriptive study | Flexor spasms (1/1) |
| Himmelreich et al.^130^ | 2022 | Germany | Not reported | Male (2) | 2 | EEG  (Neither EEG duration nor whether sleep was obtained were reported) | Not reported | Not reported | Descriptive study | Myoclonic jerks (1/2)  Mixed flexor/extensor spasms (1/2) |
| Sakpichaisakul et al.^131^ | 2022 | Thailand | Not reported | Female (1) | 1 | EEG  (Neither EEG duration nor whether sleep was obtained were reported) | 5 months | 5 months | Descriptive study | Flexor spasms (1/1) |
| Wang et al.^132^ | 2022 | China | Asian (1) | Male (1) | 1 | EEG  (Neither EEG duration nor whether sleep was obtained were reported) | 4 months | 6 months | Descriptive study | Mixed (flexor/extensor) spasms (1/1)  Head nodding (1/1) |
| Sawhney et al.^133^ | 2023 | India | Not reported | Male (1) | 1 | EEG  (Neither EEG duration nor whether sleep was obtained were reported) | 4 months | Not reported | Descriptive study | Myoclonic jerks (1/1)  Head nodding (1/1)  Associated crying (1/1) |
| Zhao et al.^134^ | 2023 | China | Not reported | Male (1) | 1 | EEG  (Neither EEG duration nor whether sleep was obtained were reported) | 5 months | Not reported | Descriptive study | Head nodding (1/1) |
| Zhang et al.^135^ | 2023 | China | Not reported | Male (1) | 1 | EEG  (Neither EEG duration nor whether sleep was obtained were reported) | 12 months | Not reported | Descriptive study | Head deviation (1/1)  Myoclonic jerks (1/1)  Mixed (flexor/extensor) spasms (1/1) |
| Caraballo et al.^136^ | 2024 | Argentina | Not reported | Not reported | 62 | EEG  (Neither EEG duration nor whether sleep was obtained were reported) | Mean: 9 months  Median: 9 months  Range: 2-23 months | Not reported | Descriptive study | Flexor spasms (39/62)  Asymmetric spasms (24/62)  Mixed (flexor/extensor) spasms (18/62)  Extensor spasms (5/62) |
| Eng and Nie^137^ | 2024 | U.S. | Not reported | Female (1) | 1 | EEG  (Neither EEG duration nor whether sleep was obtained were reported) | 3 months | 4 months | Descriptive study | Tonic spasms (1/1) |
| Oikawa et al.^138^ | 2024 | Japan | Not reported | Male (1) | 1 | EEG  (Neither EEG duration nor whether sleep was obtained were reported) | Not reported | 6 months | Descriptive study | Mixed (flexor/extensor) spasms (1/1) |
| Yüksel et al.^139^ | 2024 | Turkey | Not reported | Not reported | 96^a^ | EEG  (Neither EEG duration nor whether sleep was obtained were reported) | Mean: 5.13 months Median: 4.5 months | Patient 1: 5.5 months  Patient 2: 6 months  Patient 3: 7 months  Patient 4: 5 months  Patient 5: 5 months  Patient 6: 3 months | Descriptive study | Flexor spasms (90/96)  Mixed (flexor/extensor) spasms (4/96)  Myoclonic jerks (2/96) |
| Zhang et al.^140^ | 2024 | China | Not reported | Male (1) | 1 | EEG  (Neither EEG duration nor whether sleep was obtained were reported) | 16 months | Not reported | Descriptive study | Head nodding (1/1)  Extensor spasms (1/1) |
| ^a^This study included additional IS patients but did not report the clinical IS symptoms of these patients. Thus, we did not count those patients in the sample size. | | | | | | | | | | |

References

1. Branch CE, Dyken PR. Choroid plexus papilloma and infantile spasms. Ann Neurol. 1979; doi:10.1002/ana.410050315.

2. Kellaway P, Hrachovy RA, Frost JD, Zion T. Precise characterization and quantification of infantile spasms. Ann Neurol. 1979; doi: 10.1002/ana.410060306.

3. Willis J, Rosman NP. The Aicardi syndrome versus congenital infection: Diagnostic considerations. J Pediatr. 1980; doi:10.1016/s0022-3476(80)80808-0.

4. Curatolo P, Libutti G, Brinchi V. Infantile spasms and the CHARGE association. Dev Med Child Neurol. 1983; doi:10.1111/j.1469-8749.1983.tb13773.x.

5. Hattori H, Hayashi K, Okuno T, Temma S, Fujii T, et al. Denovo reciprocal translocation tert-(6-14)(q27-q13.3) in a child with infantile spasms. Epilepsia. 1985; doi:10.1111/j.1528-1157.1985.tb05655.x.

6. Palm L, Blennow G, Brun A. Infantile Spasms and neuronal heterotopias - a report on 6 cases. Acta Paediatr Scand. 1986; doi:10.1111/j.1651-2227.1986.tb10302.x.

7. Hrachovy RA, Frost JD, Gospe SM, Glaze DG. Infantile spasms following near-drowning - a report of 2 cases. Epilepsia. 1987; doi:10.1111/j.1528-1157.1987.tb03621.x.

8. Roos RA, Maaswinkel-Mooy PD, vd Loo EM, Kanhai HH. Congenital microcephaly, infantile spasms, psychomotor retardation, and nephrotic syndrome in two sibs. Eur J Pediatr. 1987; doi:10.1007/BF00441612.

9. Nolte R, Christen HJ, Doerrer J. Preliminary-Report of a Multi-Center Study on the West syndrome. Brain Dev. 1988; doi:10.1016/s0387-7604(88)80004-4.

10. Ichiba N. West syndrome associated with hyperlexia. Pediatr Neurol. 1990; doi:10.1016/0887-8994(90)90029-z.

11. Donat JF, Wright FS. Unusual variants of infantile spasms. J Child Neurol. 1991; doi:10.1177/088307389100600405.

12. Feng YK, Liu XQ, Sha Y, Liu PS. Infantile Spasms - a retrospective study of 105 cases. Chin Med J (Engl). 1991;104(5):416-421 (1991).

13. Carrazana EJ, Lombroso CT, Mikati M, Helmers S, Holmes GL. Facilitation of infantile spasms by partial seizures. Epilepsia. 1993; doi:10.1111/j.1528-1157.1993.tb02381.x

14. Cusmai R, Ricci S, Pinard JM, Plouin P, Fariello G, Dulac O. West syndrome due to perinatal insults. Epilepsia. 1993; doi:10.1111/j.1528-1157.1993.tb00455.x

15. Fukumizu M, Kurokawa T, Kasai H, Iso A, Yamanouchi H, et al. Unusual West syndrome with focal features and persistent suppression-burst pattern. J Epilepsy. 1993; doi:10.1016/S0896-6974(05)80005-2.

16. Fusco L, Vigevano F. Ictal Clinical electroencephalographic findings of spasms in West syndrome. Epilepsia. 1993; doi:10.1111/j.1528-1157.1993.tb00445.x

17. Koo B, Hwang PA, Logan WJ. Infantile spasms - outcome and prognostic factors of cryptogenic and symptomatic groups. Neurology. 1993; doi:10.1212/Wnl.43.11.2322.

18. Vigevano F, Fusco L, Cusmai R, Claps D, Ricci S, Milani L. The idiopathic form of West syndrome. Epilepsia. 1993; doi:10.1111/j.1528-1157.1993.tb00456.x

19. du Plessis AJ, Kramer U, Jonas RA, Wessel DL, Riviello JJ. West syndrome following deep hypothermic infant cardiac surgery. Pediatr Neurol. 1994; doi:10.1016/0887-8994(94)90111-2

20. Fois A, Tine A, Pavone L. Infantile spasms in patients with neurofibromatosis type 1. Childs Nerv Syst. 1994; doi:10.1007/BF00301086.

21. Ludvigsson P, Olafsson E, Sigurdardottir S, Hauser WA. Epidemiologic features of infantile spasms in Iceland. Epilepsia. 1994; doi:10.1111/j.1528-1157.1994.tb02514.x

22. Menezes AV, Macgregor DL, Buncic JR. Aicardi syndrome - natural-history and possible predictors of severity. Pediatr Neurol. 1994; doi:10.1016/0887-8994(94)90008-6

23. Asanuma H, Wakai S, Tanaka T, Chiba S. Brain tumors associated with infantile spasms. Pediatr Neurol. 1994; doi:10.1016/0887-8994(95)00057-m.

24. Haga Y, Watanabe K, Negoro T, Aso K, Kasai K, et al. Do ictal, clinical, and electroencephalographic features predict outcome in West syndrome? Pediatr Neurol. 1995; doi:10.1016/0887-8994(95)00157-b.

25. Bingham PM, Spinner NB, Sovinsky L, Zackai EH, Chance PF. Infantile spasms associated with proximal duplication of chromosome 15q. Pediatr Neurol. 1996; doi:10.1016/0887-8994(96)00119-1.

26. Dakshinamurty GN, Satishchandra P. Infantile spasms : electroclinical syndrome. [Neurol India](https://pubmed.ncbi.nlm.nih.gov/29542453/). 1996;44(2):67-70.

27. Kamei A, Ichinohe S, Ito M, Fujiwara T. A case of infantile spasms: epileptic apnea as partial seizures at onset. Brain Dev. 1996; doi:10.1016/0387-7604(95)00148-4.

28. Sharma AK, Jain S, Misra NK, Maheshwari MC. Infantile spasms: A prospective study in Indian children. J Epilepsy. 1996; doi:10.1016/0896-6974(96)00007-2

29. Acharya JN, Wyllie E, Luders HO, Kotagal P, Lancman M, Coelho M. Seizure symptomatology in infants with localization-related epilepsy. Neurology. 1997; doi:10.1212/Wnl.48.1.189.

30. Mitsudome A, Yasumoto S, Fukami S, Ogawa A. Kabuki make-up syndrome associated with West syndrome. Acta Paediatr Japon. 1997; doi:10.1111/j.1442-200x.1997.tb03763.x

31. Kubota T, Aso K, Negoro T, Okumura A, Natsume J, et al. Epileptic spasms preceded by partial seizures with a close temporal association. Epilepsia. 1999; doi:10.1111/j.1528-1157.1999.tb02042.x.

32. Castano G, Lyons CJ, Jan JE, Connolly M. Cortical visual impairment in children with infantile spasms. J Aapos. 2000; doi:10.1016/S1091-8531(00)70009-7.

33. Kishi T, Nejihashi Y, Kajiyama M, Ueda K. Successful zonisamide treatment for infants with hypsarrhythmia. Pediatr Neurol. 2000; doi:10.1016/S0887-8994(00)00183-1.

34. Pineda M, Vilaseca MA, Artuch R, Santos S, García González MM, et al. 3-phosphoglycerate dehydrogenase deficiency in a patient with West syndrome. Dev Med Child Neurol Suppl. 2000; doi:10.1017/S0012162200001171.

35. Sfaello I, Castelnau P, Blanc N, Ogier H, Evrard P, Arzimanoglou A. Infantile spasms and Menkes disease. Epileptic Disord. 2000; doi:10.1684/j.1950-6945.2000.tb00448.x.

36. Gaily E, Liukkonen E, Paetau R, Rekola R, Granstrom ML. Infantile spasms: diagnosis and assessment of treatment response by video-EEG. Dev Med Child Neurol. 2001; doi:10.1017/s0012162201001207.

37. Hwang YS, Korean Child Neurology S. National survey on West syndrome in Korea. Brain Dev. 2001; doi:10.1016/s0387-7604(01)00265-0.

38. Suastegui RA, De La Rosa G, Carranza JM, Gonzalez-Astiazaran A, Gorodezky C. Contribution of the MHC class II antigens to the etiology of infantile spasm in Mexican Mestizos. Epilepsia. 2001; doi:10.1046/j.1528-1157.2001.22700.x.

39. Sugai K, Fukuyama Y, Yasuda K, Fujimoto S, Ohtsu M, et al. Clinical and pedigree study on familial cases of West syndrome in Japan. Brain Dev. 2001; doi:10.1016/s0387-7604(01)00262-5.

40. Wong V. West syndrome--The University of Hong Kong experience (1970-2000). Brain Dev. 2001; doi:10.1016/s0387-7604(01)00296-0.

41. Zhou ZS, Yu WM, Yukio F, Ning ZC, Wang ZX. Clinical analysis of West syndrome associated with phenylketonuria. Brain Dev. 2001; doi:10.1016/S0387-7604(01)00260-1.

42. de Menezes MA, Rho JM. Clinical and electrographic features of epileptic spasms persisting beyond the second year of life. Epilepsia. 2002; doi:10.1046/j.1528-1157.2002.28401.x.

43. Gudino MA, Campistol J, Chavez B, Conill J, Hernandez S, Vilaseca MA. Hurler's syndrome, West's syndrome, and vitamin D-dependent rickets. J Child Neurol. 2002; doi:10.1177/088307380201700214.

44. Lortie A, Plouin P, Chiron C, Delalande O, Dulac O. Characteristics of epilepsy in focal cortical dysplasia in infancy. Epilepsy Res. 2002; doi:10.1016/s0920-1211(02)00102-x.

45. Topcu M, Saatci I, Haliloglu G, Kesimer M, Coskun T. D-glyceric aciduria in a six-month-old boy presenting with West syndrome and autistic behaviour. Neuropediatrics. 2002; doi:10.1055/s-2002-23600.

46. Yamatogi Y, Ohtahara S. Early-infantile epileptic encephalopathy with suppression-bursts, Ohtahara syndrome; its overview referring to our 16 cases. Brain Dev. 2002; doi:10.1016/s0387-7604(01)00392-8.

47. Golomb MR, Carvalho KS, Garg BP. A 9-year-old boy with a history of large perinatal stroke, infantile spasms, and high academic achievement. J Child Neurol. 2005; doi:10.1177/08830738050200051001.

48. Singhi P, Ray M. Profile of West syndrome in North Indian children. Brain Dev. 2005; doi:10.1016/j.braindev.2003.10.007.

49. Tsao CY, Wenger GD, Bartholomew DW. Cri du chat syndrome and complex karyotype in a patient with infantile spasms, hypsarrhythmia, nonketotic hyperglycinemia, and heterotopia. Am J Med Genet A. 2005; doi:10.1002/ajmg.a.30592.

50. Bahi-Buisson N, Kaminska A, Nabbout R, Barnerias C, Desguerre I, et al. Epilepsy in Menkes disease: Analysis of clinical stages. Epilepsia. 2006; doi:10.1111/j.1528-1167.2006.00432.x.

51. Kang HC, Hwang YS, Park JC, Cho WH, Kim SH, et al. Clinical and electroencephalographic features of infantile spasms associated with malformations of cortical development. Pediatr Neurosurg. 2006; doi:10.1159/000089505.

52. Kwon YS, Jun YH, Hong YJ, Son BK. Topiramate monotherapy in infantile spasm. Yonsei Med J. 2006; doi:10.3349/ymj.2006.47.4.498.

53. Lee JM, Kim AS, Lee SJ, Cho SM, Lee DS, et al. A case of infantile Alexander disease accompanied by infantile spasms diagnosed by DNA analysis. J Korean Med Sci. 2006; doi: 10.3346/jkms.2006.21.5.954.

54. Soman TB, Moharir M, Deveber G, Weiss S. Infantile spasms as an adverse outcome of neonatal cortical sinovenous thrombosis. J Child Neurol. 2006; doi:10.1177/08830738060210021001.

55. Erol I, Alehan F, Gumus A. West syndrome in an infant with vitamin B-12 deficiency in the absence of macrocytic anaemia. Dev Med Child Neurol. 2007; doi:10.1111/j.1469-8749.2007.00774.x.

56. Ito H, Mori K, Inoue N, Kagami S. A case of Kabuki syndrome presenting West syndrome. Brain Dev-Jpn. 2007; doi:10.1016/j.braindev.2006.11.005.

57. Lapatsanis P, Lapatsanis D. Pertussis vaccine and infantile spasms. Vaccine. 2007; doi:10.1016/j.vaccine.2006.08.002.

58. Mikati MA, Chaaban HR, Karam PE, Krishnamoorthy KS. Brain malformation and infantile spasms in a SCAD deficiency patient. Pediatr Neurol. 2007; doi:10.1016/j.pediatrneurol.2006.08.008.

59. Yamamoto H, Fukuda M, Miyamoto Y, Murakami H, Kamiyama N. A new trial liposteroid (dexamethasone palmitate) therapy for intractable epileptic seizures in infancy. Brain Dev. 2007; doi:10.1016/j.braindev.2006.12.002.

60. Imataka G, Kuwashima S, Hashimoto T, Yamanouchi H, Arisaka O. A Case of schizencephaly type I associated with West syndrome that responded to ACTH treatmen. DJMS. 2007;31(1):57-61.

61. Kuzmanić-Šamija R, Rešić B, Tomasović M, Pandurić DG, Lozić B, Lozić M, Resić J. West syndrome with periventricular leukomalacia: Ten-year clinical study. Coll Antropol. 2008;32(1):105-111.

62. Poirier K, Eisermann M, Caubel I, Kaminska A, Sylviane P, et al. Combination of infantile spasms, non-epileptic seizures and complex movement disorder: A new case of ARX-related epilepsy. Epilepsy Res. 2008; doi:10.1016/j.eplepsyres.2008.03.019.

63. Wallerstein R, Sugalski R, Cohn L, Jawetz R, Friez M. Expansion of the ARX spectrum. Clin Neurol Neurosur. 2008; doi:10.1016/j.clineuro.2008.03.007.

64. Ruggieri M, Iannetti P, Clementi M, Polizzi A, Incorpora G, et al. Neurofibromatosis type 1 and infantile spasms. Child Nerv Syst. 2009; doi:10.1007/s00381-008-0706-5.

65. Ibrahim S, Gulab S, Ishaque S, Saleem T. Clinical profile and treatment of infantile spasms using vigabatrin and ACTH - a developing country perspective. Bmc Pediatr. 2010; doi:10.1186/1471-2431-10-1.

66. Kumaran A, Kar S, Kapoor RR, Hussain K. The Clinical Problem of Hyperinsulinemic Hypoglycemia and Resultant Infantile Spasms. Pediatrics. 2010; doi:10.1542/peds.2009-2775.

67. Marashly A, Riel-Romero RM, Ursin S, Ghawi H. Infantile spasms associated with 5q14.3 deletion. J La State Med Soc. 2010;162(4):223-6.

68. Caraballo RH, Ruggieri V, Gonzalez G, Cersósimo R, Gamboni B, Rey A, et al. Infantile spams without hypsarrhythmia: A study of 16 cases. Seizure. 2011; doi:10.1016/j.seizure.2010.11.018.

69. Fujita Y, Imai Y, Ishii W, Endo A, Arakawa C, et al. Improvement of intractable childhood epilepsy following acute viral infection. Brain Dev. 2011; doi:10.1016/j.braindev.2010.01.002.

70. Khreisat WH. Clinical profile of infants with hypsarrhythmia. Acta Inform Med. 2011; doi:10.5455/aim.2011.19.149-152.

71. Lujic L, Bosnjak VM, Delin S, Duranovic V, Krakar G. Infantile spasms in children with Down syndrome. Coll Antropol. 2011;35(1):213-8.

72. Melani F, Mei D, Pisano T, Savasta S, Franzoni E, et al. CDKL5 gene-related epileptic encephalopathy: electroclinical findings in the first year of life. Dev Med Child Neurol. 2011; doi:10.1111/j.1469-8749.2010.03889.x.

73. Mizuno T, Sasaki M, Komaki H, Sakuma H, Saito Y, Nakagawa E, et al. A case of congenital axonal neuropathy associated with West syndrome. Brain Dev. 2011; doi:10.1016/j.braindev.2010.11.009.

74. Mordekar SR, Rittey CD, Connolly DJ, Baxter PS. Reversible basal ganglia signal changes associated with vigabatrin treatment in infants with epilepsy. J Pediatr Neurol. 2011; doi:10.3233/JPN-2012-0522.

75. Saito Y, Kubota M, Kurosawa K, Ichihashi I, Kaneko Yuu, et al. Polymicrogyria and infantile spasms in a patient with 1p36 deletion syndrome. Brain Dev. 2011; doi:10.1016/j.braindev.2010.07.004.

76. Taddio A, Bersanini C, Basile L, Fontana M, Ventura A. Gastroesophageal reflux disease at any cost: a dangerous paediatric attitude. Acta Paediatr. 2011; doi:10.1111/j.1651-2227.2011.02315.x.

77. Auvin S, Hartman AL, Desnous B, Moreau AC, Alberti C, et al. Diagnosis delay in West syndrome: misdiagnosis and consequences. Eur J Pediatr. 2012; doi:10.1007/s00431-012-1813-6.

78. Malik MA, Tarrar MA, Qureshi AO, Zia-ur-Rehman M. Clinical Spectrum of Infantile Spasm at Presentation. J Coll Physici. 2012;22(1):31-34.

79. Ricard-Mousnier B, Dorfmuller G, Fohlen M, Jeanguilaume, C, Nguyen S, et al. Late-onset epileptic spasms may be cured by focal cortical resective surgery. Epileptic Disord. 2012; doi:10.1684/epd.2012.0523.

80. Singh P, Goraya JS, Saggar K, Ahluwalia A. Aicardi syndrome. Singap Med J. 2012;53(7):E153-E155.

81. Writzl K, Primec ZR , Stražišar BG, Osredkar D, Pečarič-Meglič N, et al. Early onset West syndrome with severe hypomyelination and coloboma-like optic discs in a girl with SPTAN1 mutation. Epilepsia. 2012; doi:10.1111/j.1528-1167.2012.03437.x.

82. Bayram E, Topcu Y, Akinci G, Hiz S, Cakmakci H. Aicardi syndrome in two Turkish children. Ann Saudi Med. 2013; doi:10.5144/0256-4947.2012.01.7.1545.

83. Kaushik JS, Patra B, Sharma S, Yadav D, Aneja S. Clinical spectrum and treatment outcome of West syndrome in children from Northern India. Seizure. 2013; doi:10.1016/j.seizure.2013.04.014.

84. Lacaze E, Gruchy N, Penniello-Valette MJ, Plessis G, Richard N, et al. De Novo 15q13.3 Microdeletion with cryptogenic West syndrome. Am J Med Genet A. 2013; doi:10.1002/ajmg.a.36085.

85. Mangano S, Nardello R, Tripi G, Giordano G, Spitaleri C, et al. West syndrome followed by juvenile myoclonic epilepsy: a coincidental occurrence? Bmc Neurol. 2013; doi:10.1186/1471-2377-13-48.

86. Alrifai MT, AlShaya MA, Abulaban A, Alfadhel M. Hereditary neurometabolic causes of infantile spasms in 80 children presenting to a tertiary care center. Pediatr Neurol. 2004; doi:10.1016/j.pediatrneurol.2014.05.015.

87. Çelik T, Ünalp A, Berksoy EA, Yılmaz Ü, et al. Vitamin B12 deficiency in a child presenting with epileptic spasms. J Pediatr Neurol. 2014; doi:10.3233/Jpn-140641.

88. Holder JL, Agadi S, Reese W, Rehder C, Quach MM. Infantile spasms and hyperekplexia associated with isolated sulfite oxidase deficiency. Jama Neurol. 2014; doi:10.1001/jamaneurol.2013.5083.

89. Pontrelli G, Cappelletti S, Claps D, Sirleto P, Ciocca L, et al. Epilepsy in patients with duplications of chromosome 14 harboring FOXG1. Pediatr Neurol. 2014; doi:10.1016/j.pediatrneurol.2014.01.022.

90. Sukhudyan BG, Dimova PS, Capuano A, Vigevano F. Dyskinesia as a new adverse effect of hormonal treatment in West syndrome. Epileptic Disord. 2014; doi:10.1684/epd.2014.0628.

91. Vega YH, Kaliakatsos M, U-King-Im JM, Lascelles K, Lim M. Reversible vigabatrin-induced life-threatening encephalopathy. JAMA Neurol. 2014; doi:10.1001/jamaneurol.2013.1858.

92. Ciccone O, Kabwe A, Boniver C. Lennox-gastaut syndrome and behavioral disorder: A case report of unrecognized epilepsy in infancy. J Pediatr Epilepsy. 2015; doi:10.1055/s-0035-1555603.

93. Gulati S, Jain P, Kannan L, Sehgal R, Chakrabarty B. The clinical characteristics and treatment response in children with West syndrome in a developing country: A retrospective case record analysis. J Child Neurol. 2015; doi:10.1177/0883073815569304.

94. Guveli BT, Cokar O, Dortcan N, Benbir G, Demirbilek V, Dervent A. Long-term outcomes in patients with West syndrome: An outpatient clinical study. Seizure. 2015; doi:10.1016/j.seizure.2015.01.001.

95. Papetti L, Garone G, Schettini L, Giordano C, Nicita F, et al. Severe early onset ethylmalonic encephalopathy with West syndrome. Metab Brain Dis. 2015; doi:10.1007/s11011-015-9707-8.

96. Xixis KI, Mikati MA. Epileptic spasms: a previously unreported manifestation of WDR45 gene mutation. Epileptic Disord. 2015; doi:10.1684/epd.2015.0784.

97. Arican P, Dundar NO, Cavusoglu D, Gungor G, Gencpinar P. An occult cause of infantile spasms: Vitamin B12 deficiency. A case report and review of literature. Neurol Asia. 2016;21(3):275-278.

98. Caraballo RH, Fortini S, Reyes G, Ruiz AC, Fuentes SVS, Ramos B. Epileptic spasms in clusters and associated syndromes other than West syndrome: A study of 48 patients. Epilepsy Res. 2016; doi:10.1016/j.eplepsyres.2016.03.006.

99. Caraballo RH, Reyes G, Falsaperla R, Ramos B, et al. Epileptic spasms in clusters with focal EEG paroxysms: A study of 12 patients. Seizure. 2016; doi:10.1016/j.seizure.2016.01.012.

100. Lee HH, Hur YJ. Glucose transport 1 deficiency presenting as infantile spasms with a mutation identified in exon 9 of SLC2A1. Korean J Pediatr. 2016; doi:10.3345/kjp.2016.59.11.S29.

101. Raviglione F, Conte G, Ghezzi D, Parazzini C, Righini A, et al. Clinical findings in a patient with FARS2 mutations and early-infantile-encephalopathy with epilepsy. Am J Med Genet A. 2016; doi:10.1002/ajmg.a.37836.

102. Yilmaz S, Tekgul H, Serdaroglu G, Akcay A, Gokben S. Evaluation of ten prognostic factors affecting the outcome of West syndrome. Acta Neurol Belg. 2016; doi:10.1007/s13760-016-0611-8.

103. Benitez V, Manley PE, Goumnerova LC, Harini C, Ullrich NJ. Brain tumors and epileptic spasms: Natural history and outcomes. Epilepsy Behav. 2017; doi:10.1016/j.yebeh.2017.07.036.

104. Caraballo RH, Falsaperla R, Gutierrez R, Reyes G. Single-epileptic spasms with or without hypsarrhythmia: a study of 16 patients (vol 6, pg e1, 2017). J Pediatr Epilepsy. 2017; doi:10.1055/s-0037-1603912.

105. Gul Mert G, Herguner MO, Incecik F, Altunbasak S, Sahan D, Unal I. Risk factors affecting prognosis in infantile spasm. Int J Neurosci. 2017; doi:10.1080/00207454.2017.1289379.

106. Kasinathan A, Padmanabh H, Gupta K, Sankhyan N, Singh P, Singhi P. Unusual cause of West syndrome. J Pediatr Neurosci. 2017; doi:10.4103/jpn.JPN_24_17.

107. Kobayashi Y, Ishikawa N, Tani H, Fujii Y, Kobayashi M. Recurrence of epileptic spasms as reflex seizures induced by eating: A case report and literature review. Neuropediatrics. 2017; doi:10.1055/s-0037-1598111.

108. Kvernadze A, Tatishvili N, Kipiani T, Lomidze G. Characteristics of West syndrome in georgia, preliminary results of the prospective study. Georgian Med News. 2017;(272):104-109.

109. Jiang L, Gao F, Mao S, Xu J, Jiang K. Somatic KRAS mutation in an infant with linear nevus sebaceous syndrome associated with lymphatic malformations: A case report and literature review. Medicine (Baltimore). 2017; doi:10.1097/MD.0000000000008016.

110. Pereira AG, Bahi-Buisson N, Barnerias C, Boddaert N, et al. Epileptic spasms in congenital disorders of glycosylation. Epileptic Disord. 2017; doi:10.1684/epd.2017.0901.

111. Ciara E, Rokicki D, Lazniewski M, Mierzewska HB. Clinical and molecular characteristics of newly reported mitochondrial disease entity caused by biallelic PARS2 mutations. J Hum Genet. 2018; doi:10.1038/s10038-017-0401-z.

112. Kulsoom S, Ibrahim SH, Jafri SK, Moorani KN, Anjum M. Infantile Spasms: Clinical profile and treatment outcomes. Pak J Med Sci. 2018; doi:10.12669/pjms.346.15869.

113. Ben Abdelaziz R, Ben Chehida A, Lamouchi M, Messaoud SB, Mohamed DA, Boudabous H. Factors predictive of prognosis of infantile spasms. A retrospective study in a low-income country. Arch Pediatr. 2019; doi:10.1016/j.arcped.2018.10.003.

114. Chong PF, Matsukura M, Fukui K, Watanabe Y, Matsumoto N, Kira R. West syndrome in an infant with vitamin b12 deficiency born to autoantibodies positive mother. Front Pediatr. 2019; doi:10.3389/fped.2019.00531.

115. Li H, Wang B, Shan L, Du L, Jia F. Spontaneous remission of West syndrome following a human herpesvirus 7 infection in a Chinese infant: A case report. Medicine (Baltimore). 2019; doi:10.1097/MD.0000000000016441.

116. Yoshida N, Arai Y, Kasai Y, Ohtomo Y, Niijima S, Shimizu T. West syndrome associated with glycogen storage disease type 1b. Epilepsy & Seizure. 2019; doi:10.3805/eands.11.46.

117. Barbarrosa EP, Ferrer IDP, Tovani-Palone MR. West syndrome: clinical characteristics, therapeutics, outcomes and prognosis. Electron J Gen Med. 2020; doi:10.29333/ejgm/7800.

118. Charkhand B, Liu N, Barrett KT, Al-Hertani W, Scantlebury MH. An unusual case of infantile spasms due to a pathogenic variant in the MECP2 gene. J Pediatr Neurol. 2020; doi:10.1055/s-0039-1683436.

119. Desprairies C, Valence S, Maurey H, Helal SI, Weckhuysen S, Soliman H. Three novel patients with epileptic encephalopathy due to biallelic mutations in the PLCB1 gene. Clin Genet. 2020; doi:10.1111/cge.13696.

120. McFarlane MT, Wright T, Mccoy B, Snead OC, Westall CA. Retinal defect in children with infantile spasms of varying etiologies An observational study (vol 94, e575, 2020). Neurology. 2020; doi:10.1212/Wnl.0000000000009279.

121. Romano C, Ferranti S, Mencarelli MA, Longo I, Renieri A, Grosso S. 17p13.3 microdeletion including YWHAE and CRK genes: towards a clinical characterization. Neurol Sci. 2020; doi:10.1007/s10072-020-04424-3.

122. Sharawat IK, Panda PK. Epileptic spasms in an infant with incontinentia pigmenti: report of a rare case with brief review of the literature. J Neurosci Rural Pra. 2020; doi:10.1055/s-0040-1709246.

123. Zhang L, Mao X, Long HY, Xiao B, Luo Z, et al. Compound heterozygous PIGS variants associated with infantile spasm, global developmental delay, hearing loss, visual impairment, and hypotonia. Front Genet. 2020; doi:10.3389/fgene.2020.00564.

124. Fukuoka M, Okazaki S, Kim K, Nukui M, Inoue T, et al. Preliminary report for Epilepsia Open A case of West syndrome with severe global developmental delay and confirmed KIF5A gene variant. Epilepsia Open. 2021; doi:10.1002/epi4.12431.

125. Sun YL, Wan L, Yan HM, Li ZC, Yang G. Phenotypic and genotypic characterization of NPRL2-related epilepsy: two case reports and literature review. Front Neurol. 2021; doi:10.3389/fneur.2021.780799.

126. Crippa ACS, Bayer DL, Souza LP, Franklin GL. Adrenocorticotropic Hormone (ACTH)-induced dyskinesias in infantile spasms: a video case report. Am J Case Rep. 2022; doi:10.12659/ajcr.935349.

127. Haneder C, Stark B, Peherstorfer A, Groppel G. Focal signs in infantile spasms. Seizure. 2022; doi:10.1016/j.seizure.2022.02.008.

128. Sabapathy T, Bansal R, Bojorquez L, Salley B, Gianakon JG, et al. Missed opportunities and the impact of the pandemic. J Dev Behav Pediatr. 2022; doi:10.1097/Dbp.0000000000001065.

129. Madan S, Chaudhuri Z. Persistent hyperplastic primary vitreous in a child with incontinentia pigmenti and infantile spasms. Oman J Ophthalmol. 2022; doi:10.4103/ojo.ojo_194_21.

130. Himmelreich N, Dimitrov B, Zielonka M, Hüllen A, Hoffmann GF, et al. Missense variant c.1460 T > C (p.L487P) enhances protein degradation of ER mannosyltransferase ALG9 in two new ALG9-CDG patients presenting with West syndrome and review of the literature. Mol Genet Metab. 2022; doi:10.1016/j.ymgme.2022.06.005.

131. Sakpichaisakul K, Boonkrongsak R, Lertbutsayanukul P, Lemwimangsa N, Klumsathian S, et al. Epileptic spasms related to neuronal differentiation factor 2 (NEUROD2) mutation respond to combined vigabatrin and high dose prednisolone therapy. Bmc Neurol. 2022; doi:10.1186/s12883-022-02992-9.

132. Wang H, Zhu Y, Cao D, Chen H, Ding X, et al. Successful medical treatment of West syndrome with a KCNA2 variant: A case report. Acta Epileptol. 2022; doi:10.1186/s42494-021-00069-7.

133. Sawhney S, Vagha K, Gomase S, Lohiya S, Hampe P, et al. The early presentation of a classic case of tuberous sclerosis: A case report. Cureus. 2023; doi:10.7759/cureus.47241.

134. Zhao S, Lian R, Jin L, Li M, Jia T, et al. Clinical and genetic analysis of infants with pontocerebellar hypoplasia type 6 caused by variations. Epilepsia Open. 2024; doi:10.1002/epi4.12862.

135. Zhang S, Lin S, Wang W, Gan Y, Wang C, et al. Developmental and epileptic encephalopathy 44 due to compound heterozygous variants in the UBA5 gene: a case report. Acta Epileptol. 2023; doi:10.1186/s42494-023-00139-y.

136. Caraballo RH, Gallo A, Reyes G, Flores G, Martín E, et al. Epileptic spasms in clusters without hypsarrhythmia in infancy and childhood: A single age-dependent type of epilepsy or well-defined epileptic syndrome? Epilepsy Res. 2024; doi:10.1016/j.eplepsyres.2024.107354.

137. Eng NY, Nie DA. Infantile epileptic spasms syndrome in a child with lissencephaly associated with de novo PAFAH1B1 variant and coincidental CMV infection. Epilepsy Behav Rep. 2024; doi:10.1016/j.ebr.2024.100664.

138. Oikawa S, Yamaguchi H, Hanafusa H, Ye MJ. Treatment options for infantile spasms syndrome with scn8a: A case report and literature review. J Pediatr Epilepsy. 2024; doi:10.1055/s-0043-1778011.

139. Yüksel MF, Doğulu N, Yıldırım M, Köse E, Bektaş Ö, Eminoğlu FT, Teber S. Metabolic etiologies in children with infantile epileptic spasm syndrome: Experience at a tertiary pediatric neurology center. Brain Dev. 2024; doi:10.1016/j.braindev.2024.03.003.

140. Zhang Q, Zou L, Lu Q, Wang Q, Dun S, Wang J. Genetic variant reanalysis reveals a case of Sandhoff disease with onset of infantile epileptic spasm syndrome. Acta Epileptol. 2024; doi:10.1186/s42494-024-00149-4.
